# Novel 24-h ovine model of brain death to study the profile of the endothelin axis during cardiopulmonary injury

**DOI:** 10.1186/s40635-015-0067-9

**Published:** 2015-11-24

**Authors:** Ryan P. Watts, Izabela Bilska, Sara Diab, Kimble R. Dunster, Andrew C. Bulmer, Adrian G. Barnett, John F. Fraser

**Affiliations:** Critical Care Research Group, The Prince Charles Hospital, Chermside, Queensland Australia; Heart Foundation Research Centre, Griffith Health Institute, Griffith University, Southport, Queensland Australia; Institute of Health and Biomedical Innovation, Queensland University of Technology, Brisbane, Queensland Australia; University of Queensland, Brisbane, Queensland Australia; Royal Brisbane and Women’s Hospital, Herston, Queensland Australia

**Keywords:** Brain death, Organ transplantation, Haemodynamics, Pulmonary circulation, Ventricular Function, Right, Endothelin-1, Receptors, Endothelin, Sheep

## Abstract

**Background:**

Upregulation of the endothelin axis has been observed in pulmonary tissue after brain death, contributing to primary graft dysfunction and ischaemia reperfusion injury. The current study aimed to develop a novel, 24-h, clinically relevant, ovine model of brain death to investigate the profile of the endothelin axis during brain death-associated cardiopulmonary injury. We hypothesised that brain death in sheep would also result in demonstrable injury to other transplantable organs.

**Methods:**

Twelve merino cross ewes were randomised into two groups. Following induction of general anaesthesia and placement of invasive monitoring, brain death was induced in six animals by inflation of an extradural catheter. All animals were supported in an intensive care unit environment for 24 h. Animal management reflected current human donor management, including administration of vasopressors, inotropes and hormone resuscitation therapy. Activation of the endothelin axis and transplantable organ injury were assessed using ELISA, immunohistochemistry and standard biochemical markers.

**Results:**

All animals were successfully supported for 24 h. ELISA suggested early endothelin-1 and big endothelin-1 release, peaking 1 and 6 h after BD, respectively, but there was no difference at 24 h. Immunohistochemistry confirmed the presence of the endothelin axis in pulmonary tissue. Brain dead animals demonstrated tachycardia and hypertension, followed by haemodynamic collapse, typified by a reduction in systemic vascular resistance to 46 ± 1 % of baseline. Mean pulmonary artery pressure rose to 186 ± 20 % of baseline at induction and remained elevated throughout the protocol, reaching 25 ± 2.2 mmHg at 24 h. Right ventricular stroke work increased 25.9 % above baseline by 24 h. Systemic markers of cardiac and hepatocellular injury were significantly elevated, with no evidence of renal dysfunction.

**Conclusions:**

This novel, clinically relevant, ovine model of brain death demonstrated that increased pulmonary artery pressures are observed after brain death. This may contribute to right ventricular dysfunction and pulmonary injury. The development of this model will allow for further investigation of therapeutic strategies to minimise the deleterious effects of brain death on potentially transplantable organs.

**Electronic supplementary material:**

The online version of this article (doi:10.1186/s40635-015-0067-9) contains supplementary material, which is available to authorized users.

## Background

After brain death (BD), the lungs are particularly susceptible to injury in the peri-transplant period secondary to direct trauma, soiling with blood or gastric contents, iatrogenic injury, infection and inflammation [1–3]. Details of the specific mechanisms of catecholamine- and cytokine-induced donor organ injury after BD are yet to be fully elucidated [2, 4, 5]. Peri-transplant injury contributes to the ongoing shortage of transplantable lungs; this is highlighted by American data reporting an average rate of lungs transplanted per donor of 0.37 [6].

Endothelins, their precursors, receptors and associated signalling pathways are collectively referred to as the endothelin axis [7, 8]. Endothelin-1 (ET-1) is a potent vasoconstrictor, smooth muscle cell and fibroblast mitogen and a stimulator of inflammatory cell infiltration [9–11]. Once released, ET-1 stimulates matrix metalloproteinase (MMP) expression in pulmonary tissue, resulting in protein hydrolysis and interstitial oedema [2, 8]. Our group first demonstrated that the endothelin axis was upregulated after BD in rats, and that this correlated with pulmonary injury [8]. Upregulation of endothelin receptors “primes” the lungs for post-transplant injury [2] and may partly explain the relationship between endothelin expression and primary graft dysfunction that has been observed in human lung allograft recipients [12].

Haemodynamic instability has limited the duration of previous BD animal studies and supportive measures used to extend these models to clinically relevant timeframes are difficult to apply to small animals [13–15]. Interventions utilised in human BD donors, such as fluid or vasoactive agent administration, may have significant effects on genomic expression of inflammatory mediators [16, 17], further limiting the ability of small animal models to replicate comprehensive, modern, intensive care monitoring and management. To begin to address these issues, porcine models have been extended to 24 h [18, 19]. Zhai et al*.* investigated hepatic injury after BD in BaMa miniature pigs [18]. Although this is a valid extended model, the animals were small compared to humans (average of 25 kg), and the use of other clinically relevant interventions, such as vasopressors, inotropes and hormone resuscitation, were not reported. The model published by Sereinigg et al*.* was developed to more closely reflect clinical experience with BD donors, including the use of vasoactive agents, however this publication did not specifically include a control group for comparison [19].

No animal model can absolutely replicate all aspects of human pathophysiology [20]. For example, controversy exists regarding rodent modelling of human disease, with evidence both supporting and refuting similarities of inflammatory genomic responses to injury between the two species [16, 21]. Both pigs and sheep have been effectively utilised as large animal models of human pathology, with each offering notable benefits [22, 23]. Ovine models have been highlighted as particularly suitable for investigating human lung disease [17, 23–25]. Furthermore, sheep models have provided detailed insight into the endothelin axis and its contribution to pulmonary haemodynamics, as well as the role of ET-1 in lung inflammation [23, 26–28]. Therefore, based on these considerations, we have developed a 24-h ovine model to investigate the role of the endothelin axis in BD-related pulmonary inflammation. Additionally, the results of comprehensive investigation of the effects of BD on ovine haemodynamics and systemic markers of transplantable organ injury are presented.

## Methods

### Ethics approval

This study was conducted with the approval of the Queensland University of Technology Animal Ethics Committee, approval number 0900000319. All experiments were performed in accordance to NHMRC *Australian Code of Practice for the Care and Use of Animals for Scientific Purposes* and the *Animal Care and Protection Act 2001 (QLD)*.

### Animal management

Twelve merino cross ewes were randomly allocated to groups of six animals each (BD vs control) using Statmate (GraphPad Software, La Jolla, CA). Initial surgical preparation was the same in all animals. A comprehensive description of the animal management protocol can be found in Additional file 1, whilst Table 1 lists the details of the medications used in this study. After fasting, the external jugular veins were cannulated, general anaesthesia was induced with midazolam and alfaxalone, and all animals were intubated. Pulmonary arterial and peripheral arterial catheters were placed. Intracranial access was obtained through a burr-hole midway between the midline and lateral edge of the cranium, rostral to the animal’s horn base, and an intracranial pressure monitor was introduced. This was designated as the protocol start time (PST) in non-BD animals.

**Table 1:** Medications for the protocol

Drug		Bolus	Initial infusion rate	Notes
Anaesthetic induction				
	Lignocaine 1 %	3–5 mL subcutaneously		Over central venous access insertion sites
	Buprenorphine	300 mcg		Administered six hourly during protocol
	Midazolam	0.5 mg/kg		
	Alfaxalone	3 mg/kg		If further boluses needed, dosed at 0.5 mg/kg
Anaesthetic maintenance				
	Alfaxalone		6 mg/kg/h	Adjusted to surgical plane
	Ketamine		3 mg/kg/h	Adjusted to surgical plane
	Midazolam		0.25 mg/kg/h	Used only if required (if alfaxalone exceeded 250 mg/h)
Antimicrobial prophylaxis				
	Cefalotin	1000 mg		
	Gentamicin	40 mg		
Fluid management				
	Hartmann’s solution	10–20 mL/kg	2 mL/kg/h	Titrated to CVP 8–12 mmHg. Boluses if needed for low urine output (<0.5 mL/kg/h) or hypotension (MAP < 60 mmHg). Initial fluid of choice
	Normal saline 0.9 %	10–20 mL/kg	1–2 mL/kg/h	Boluses if needed for low urine output (UO < 0.5 mL/kg/h) or hypotension (MAP < 60 mmHg)
	Dextrose 5 % or dextrose 4 % in saline 0.18 %	10–20 mL/kg	1–2 mL/kg/h	Utilised for hypoglycaemia (BSL < 6 mmol/L)
Vasopressors, inotropes and cardiovascular support				
	Metaraminol	0.5–1 mg		Utilised in emergency situations for hypotension only
	Atropine	600 mcg		Utilised in emergency situations for bradycardia (HR < 60 bpm) only
	Noradrenaline		0.05 mcg/kg/min	Adjusted to MAP > 60 mmHg
	Dopamine		5 mcg/kg/min	Adjusted to MAP > 60 mmHg
	Isoprenaline		0.5 mcg/min	Adjusted to MAP > 60 and HR > 60 bpm. Utilised only if considered bradycardia as cause of hypotension
	Glyceryl trinitrate		0.1 mg/h	For hypertension (SBP > 180 mmHg) if necessary
	Amiodarone		5 mg/kg over 2 h	Infusion for appropriate dysrhythmias (e.g. atrial fibrillation) if necessary. Could be repeated
Hormonal management				
	Insulin	10–20 U	0.5 U/h	Bolus for BSL > 16 mmol/L. Infusion adjusted to BSL 6–10 mmol/L, tested hourly once infusion commenced
	Dextrose 50 %	25 mL		For management of hypoglycaemia (BSL < 3.5 mmol/L). Please also note that dextrose 5 % could be used for ongoing maintenance per above
	Desmopressin	4 mcg		If urine output >300 mL/h for two consecutive hours
Hormone resuscitation at 12 h				
	Vasopressin	1 U	0.5–4.0 U/h (initial dose 2.0 U/h)	Adjusted to SVR 800–1200 dyn.s.cm^−5^
	Liothyronine	4 mcg	3 mcg/h	
	Methylprednisolone	15 mg/kg		
Electrolyte management				
	Potassium chloride		10–40 mmol/h	Adjusted to potassium 3.5–5.0 mmol/L
	Calcium chloride 10 %	6.8 mmol		Administered to keep ionised calcium > 1.05 mmol/L
	Magnesium sulphate	10–20 mmol		Allowed for management of dysrhythmias (e.g. atrial fibrillation)
Euthanasia				
	Sodium pentobarbitone	100 mg/kg		

Not all agents were used. Agents listed include medications that were able to be used in the case of predetermined outcomes or complications

Another burr hole was created on the contralateral side in animals allocated to BD, followed by the extradural placement of a 16 Fr Foley catheter. Brain death was induced by normal saline inflation of the catheter to increase intracranial pressure (ICP) above the mean arterial pressure (MAP) for greater than 30 min [29]. Commencement of inflation served as the BD induction time (BIT). Confirmation of brain death was achieved by continuously negative cerebral perfusion pressure (defined as MAP-ICP) for greater than 30 min, loss of pupillary and corneal reflexes and lack of respiratory efforts. Protocol start time was deemed once BD was confirmed in animals allocated to this group. Due to variability in duration required for induction and confirmation of BD, haemodynamic results are reported as time from BIT. Haemodynamic deterioration was managed with intravenous fluid and vasopressors or inotropes as appropriate.

Twelve hours after PST, hormone therapy was commenced with vasopressin, methylprednisolone and liothyronine in all animals. This time point was chosen to reflect the clinical realities of delays in diagnosis and confirmation of brain death, family consent for organ transplantation, and the change from lifesaving to organ preserving treatment [30]. After completion of the 24-h protocol, the animals were sacrificed using sodium pentobarbitone.

### Sample retrieval and storage

Blood was collected from the peripheral arterial line at baseline (prior to BIT), 1, 6, 12, 18 and 24 h after confirmation of BD. Blood samples were then centrifuged, supernatant transferred into vials (Eppendorf, North Ryde, Australia) and stored at −80 °C until analysis. After animals were euthanised, the lungs were removed en bloc and samples taken from both lower lobes. These were fixed in 10 % phosphate-buffered formalin, embedded in paraffin and mounted on slides for histological analysis.

### Histological and tissue analysis

Samples were taken from the right lower lobe to assess for wet:dry weight ratio, as an indicator of inflammatory oedema. These were dehydrated in an oven at 45 °C for 48 h, at which time they were reweighed and the ratio calculated.

Haematoxylin and eosin staining of lung specimens was performed to allow morphologic assessment of tissue samples. Inflammation was graded semi-quantitatively as previously reported [8].

Immunohistochemical staining was employed to assess the patency of the endothelin axis using standard methods (see also Additional file 1) [31]. Monoclonal anti-ET-1 (Sigma Aldrich, St Louis, MO), polyclonal anti-ET_R_A, anti-ET_R_B and anti-MMP-2 (Merck Millipore, Billerica, MA), polyclonal anti-MMP-9 (Biorbyt, San Francisco, CA) and polyclonal anti-TIMP-1 (tissue inhibitor of metalloproteinase-1) and anti-TIMP-2 (Bioss, Woburn, MA) were selected as primary antibodies.

Immunohistochemistry and histological scoring was performed independently by two investigators (RW and IB) and results compared. Disagreements in scoring were resolved by using the lowest score (i.e. indicating less injury). Slides were assessed in random order and the assessors were blinded to group of allocation.

### ELISA

Systemic concentrations of ET-1 and pro-endothelin-1 (big ET-1) were assessed in EDTA plasma using commercially available sandwich ELISA kits (BiomedicaGruppe, Austria). Absorbance was read at 450 nm with reference 630 nm on a 96-well plate spectrophotometer (FluoStar Omega, BMG LabTech, Germany). The results from one animal in the BD group were excluded due to technical reasons preventing accurate spectrophotometric analysis.

### Biochemical analysis

Biomarkers of organ injury were assayed using the COBAS Integra 400 chemical analyser (Roche Diagnostics, Dee Why, Australia), following manufacturer’s instructions. Reagent cassettes were calibrated using the calibrator for automated systems (CFAS, Roche Diagnostics). Precision and accuracy of assays were confirmed using standard quality controls (Precinorm Clin Chem Multi 1 and 2, Roche Diagnostics). All tests were performed in duplicate, averaged and compared to CFAS in order to interpolate sample concentrations.

### Statistical analysis

Analysis of biochemical data was performed using Prism 6 (GraphPad Software Inc., USA). All regression analyses were conducted using R software (www.r-project.org). A two-sided statistical significance level of <0.05 was adopted. Results are reported as mean ± standard deviation. Two-way repeated-measures analysis of variance (ANOVA) was used to test for significant differences in dependent variables. Student’s *t* test was used to compare changes in physiological variables at specified time points. Fisher’s exact test was used to compare semi-quantitative assessment of tissue samples. For continuous physiological variables, a regression model was used to examine the changes in variables over time. A mixed model with a random intercept for each sheep to account for repeated responses from the same animal was used [32].

## Results

All 12 animals survived the 24-h protocol and induction of BD was successful in all animals allocated to this group. Summary tables detailing ventilation, haemodynamics, fluid balance, biochemistry and histology can be found in Additional file 1.

### Animal management and point of care testing

**Fig. 1:**
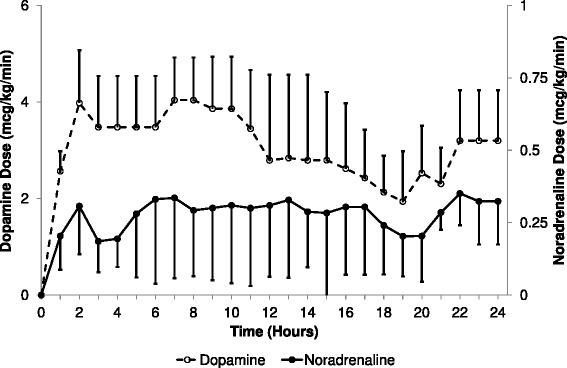
Doses of vasoactive agents administered to brain dead sheep. Mean doses of dopamine and noradrenaline administered to the brain dead animals over the duration of the study

There were no differences between the animal groups with regard to mechanical ventilation. Markers of oxygenation, P(A-a)O_2_ and PaO_2_:FiO_2_, deteriorated in BD animals over the first 2 h. Mean P(A-a)O_2_ in BD animals was 145 ± 79 mmHg (19.3 ± 105 kPa) at 1 h (66.8 ± 40 mmHg (8.9 ± 5.3 kPa, *p* < 0.001) greater than controls) and 87 ± 45 mmHg (11.5 ± 5.9 kPa) at 2 h (45.7 ± 40 mmHg (6.09 ± 5.3 kPa, *p* = 0.016) greater than controls). PaO_2_:FiO_2_ was 221 ± 81 less in BD animals (absolute value 294 ± 83, *p* < 0.001) at 1 h and 110 ± 80 less at 2 h (absolute value 432 ± 114, *p* = 0.003). These variables were thereafter similar to controls and no difference was found at 24 h (*p* = 0.56 P(A-a)O_2_ and *p* = 0.87 PaO_2_:FiO_2_). Minute ventilation was similar between the groups, with a trend towards lower PaCO_2_ in the control group at 24 h (PaCO_2_ 27 ± 4 mmHg (3.6 ± 0.5 kPa) control vs 32 ± 5 mmHg (4.3 ± 0.6 kPa) BD, *p* = 0.051). Lactate, a surrogate marker of hypoperfusion, was significantly elevated in the BD group (*p* = 0.03), reaching a peak value of 2.75 ± 3.3 mmol/L at 18 h. There was no difference in blood pH between groups (*p* = 0.85). No vasoactive agents were required in the control group, whereas all BD animals required vasoactive support (Fig. 1). Each of the six BD animals met predefined criteria for diabetes insipidus and required desmopressin. Although BD animals therefore had greater urine output and fluid administration, cumulative fluid balance at 24 h was not different between groups (2.1 ± 0.8 L control vs 2.4 ± 1.7 L BD, *p* > 0.9).

### Physiologic variables

Time to confirmation of brain death after inflation of the extradural catheter was 50 ± 22 min. During this time, the highest ICP achieved was 237 ± 79 mmHg, with a resultant CPP of −117 ± 34 mmHg. At completion of the study, ICP was 87 ± 12 mmHg with a CPP of −6.5 ± 12 mmHg.

**Fig. 2:**
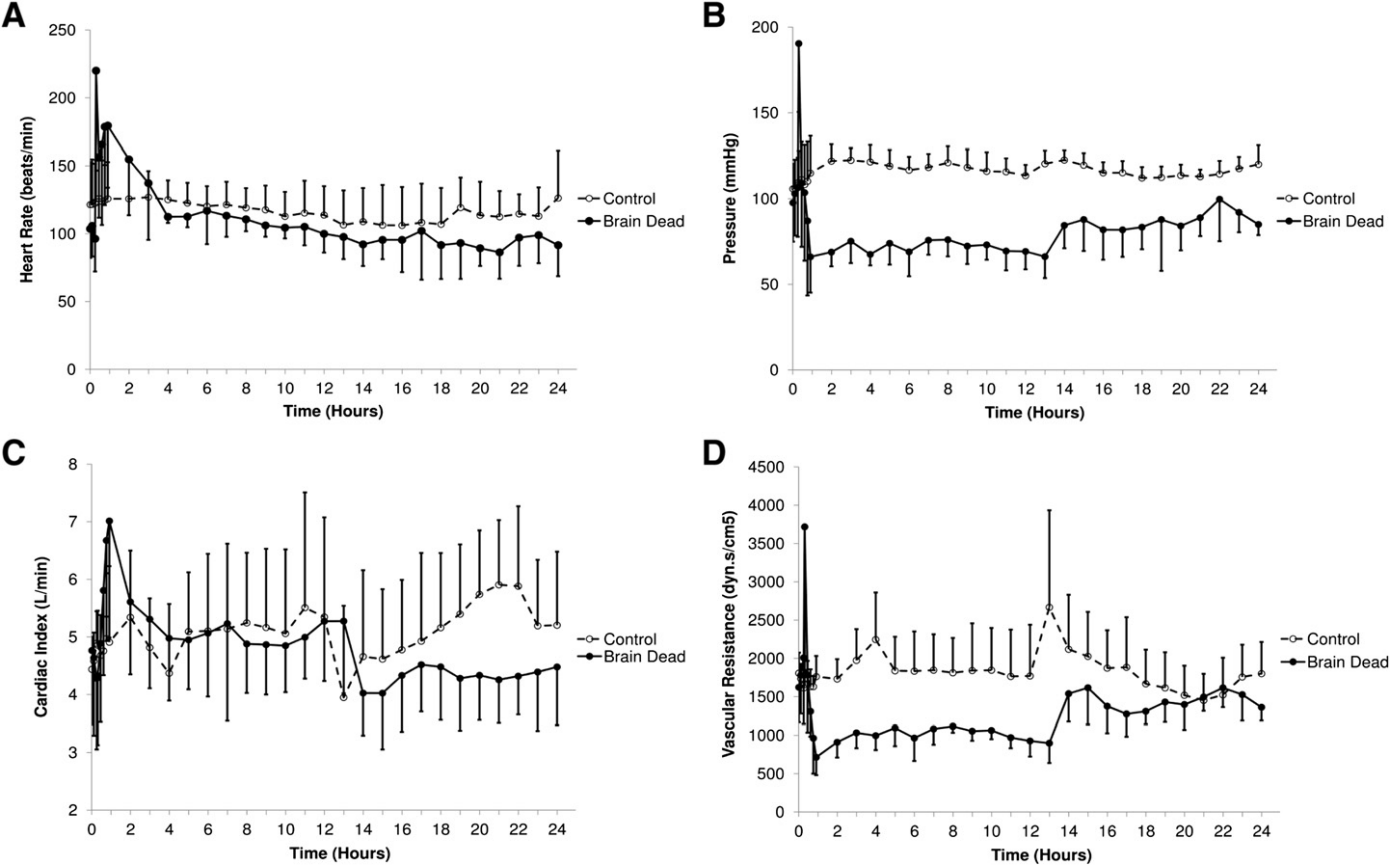
Systemic cardiovascular responses observed in brain dead and control animals during induction of BD and over 24 h. **a** Heart rate. After an early peak, heart rate decreased and was similar to controls at 24 h. **b** Mean arterial pressure increased with induction of brain death and then fell below baseline and control animals. Although some improvement occurred with administration of hormone therapy at 12 h, blood pressure remained below controls to 24 h. **c** Cardiac index was also elevated after induction of brain death but returned to control levels by 3 h. It fell to a level lower than controls after administration of hormone therapy. **d** Systemic vascular resistance index followed a similar pattern to mean arterial pressure. Brain death was induced immediately after the baseline value at time 0

Brain death caused tachycardia, hypertension and elevated cardiac output (Fig. 2a–c). Cardiac index (CI) increased primarily as a consequence of tachycardia with the stroke volume index (SVI) acutely decreasing from 45 ± 1 to 30 ± 6 mL/m^2^ (*p* < 0.001). Mean arterial pressure increased from 99 ± 3 mmHg to peak at 193 ± 40 mmHg during induction of BD, decreasing to 58 ± 2 % of baseline at 90 min after BIT (*p* < 0.001) and remaining lower than the control group at 24 h (*p* < 0.001). Systemic vascular resistance index (SVRI—Fig. 2d) increased from 1741 to 3718 dyn.s.cm^−5^ within 5 min of extradural catheter inflation, falling to 46 ± 1 % of baseline by 1 h and remaining depressed throughout the remainder of the study. After initiation of hormone resuscitation, SVRI increased to 81 ± 7 % of baseline. Cardiac index increased to a peak of 7.48 ± 2.1 L/min/m^2^ from a baseline of 4.55 ± 0.18 L/min/m^2^ 30 min after BIT and remained 14 ± 5 % above baseline until 1 h after hormonal therapy was commenced, whereby it returned to baseline levels. At 24 h, there was no statistical difference (*p* = 0.79 compared to baseline, *p* = 0.91 compared with controls). Left ventricular stroke work index (LVSWI) was significantly reduced in BD animals compared to controls (*p* < 0.001). After decreasing to 19.8 ± 0.69 g.m/m^2^/beat at 75 min post-BIT, LVSWI returned to 35.1 ± 1.3 g.m/m^2^/beat over the following 4 h. Hormonal resuscitation therapy increased LVSWI to 44.8 ± 2.8 g.m/m^2^/beat at 24 h (*p* < 0.001 compared to baseline).

**Fig. 3:**
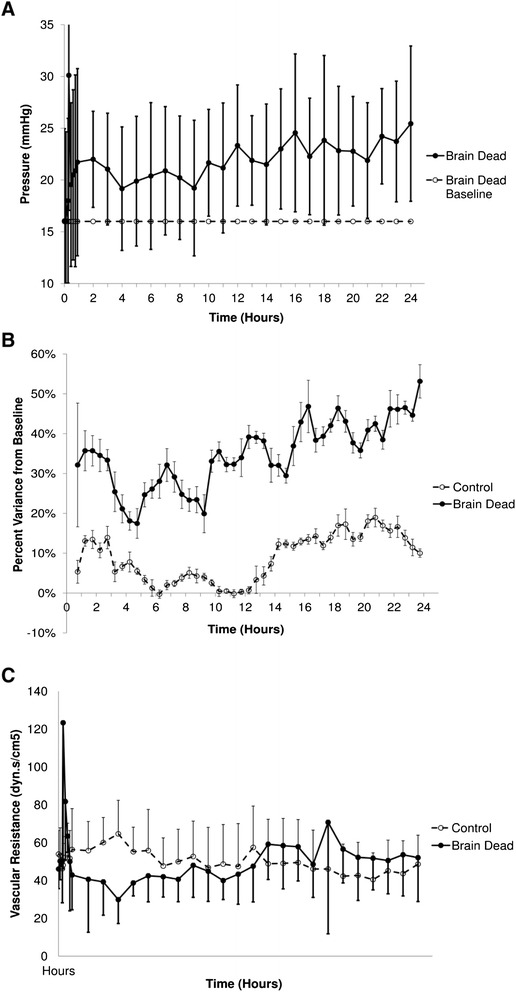
Pulmonary haemodynamic responses observed in brain dead and control animals during induction of BD and over 24 h. **a** Mean pulmonary arterial pressure peaked early after induction of brain death, increasing over the duration of the study. **b** Percent variance from baseline of mean pulmonary artery pressure demonstrates that this deviated to a greater degree over time than control animals. **c** Pulmonary vascular resistance index also demonstrated an early peak and had returned to levels consistent with controls by 24 h. Brain death was induced immediately after the baseline value at time 0

Mean pulmonary artery pressure (mPAP) peaked at 186 ± 20 % of baseline with induction of BD (*p* < 0.001), rising from 16 ± 0.2 to 30 ± 13 mmHg. After the initial peak, mPAP remained 31 ± 2 % greater than baseline 90 min after BIT (*p* < 0.001) and continued to increase for the remainder of the experiment (Fig. 3a, b). Pulmonary vascular resistance index (PVRI) increased from 50 ± 3 to 123 ± 77 dyn.s.cm^−5^ within 5 min of Foley catheter inflation (Fig. 3c), decreasing to 55 ± 4 % of baseline at 4 h after BD. The PVRI returned to baseline after initiation of hormone resuscitation and was not different from the control group at 24 h (*p* = 0.5). Right ventricular stroke work index (RVSWI) had decreased 15 min after BIT (by 1.4 ± 0.7 g.m/m^2^/beat, *p* < 0.001). However, by 30 min, this had increased to be 5.4 ± 0.01 % above the baseline of 6.5 ± 0.24 g.m/m^2^/beat (*p* = 0.01) and continued to increase to 25.9 % above baseline at the end of the study in the BD animals (*p* < 0.001).

### Histological and tissue analysis

There was no statistically significant difference in lung wet:dry ratio between groups, with an average ratio of 3.48 vs 3.39 (non-BD vs BD, *p* = 0.68).

Semi-quantitative assessment of lower lobe pulmonary samples demonstrated increased inflammation in BD animals (non-BD animals: none-mild inflammation (0 or +); BD animals: moderate-severe inflammation changes (++ or +++), including increased interstitial oedema and inflammatory cell infiltration, *p* = 0.014).

The endothelin axis was detectable by immunohistochemical staining (examples can be found in Additional file 1). Staining of ET-1 was localised to bronchiolar epithelium and perivascular smooth muscle in both BD and non-BD animals, with no appreciable difference in expression noted. With regard to the endothelin receptors, ET_R_A was stained minimally in both groups within bronchiolar epithelium and smooth muscle, and ET_R_B was well localised to airway columnar epithelium. There was no difference in expression of either receptor between groups. Overall, there were no differences in intensity of staining for MMP-2 or MMP-9 between groups, with MMP-2 slightly expressed within vascular endothelial and bronchiolar epithelial cells and MMP-9 able to be identified within bronchiolar epithelium and perivascular smooth muscle.

Low intensity of staining for TIMP-1 and -2 was observed for both groups, with no detectable difference. Staining of TIMP-1 was primarily localised within the columnar epithelia in the bronchioles with some staining within the alveolar parenchyma. Similarly to TIMP-1, TIMP-2 expression was mainly observed in bronchiolar epithelia, though some staining in the alveolar parenchyma and pulmonary blood vessels was noted.

### ELISA

**Fig. 4:**
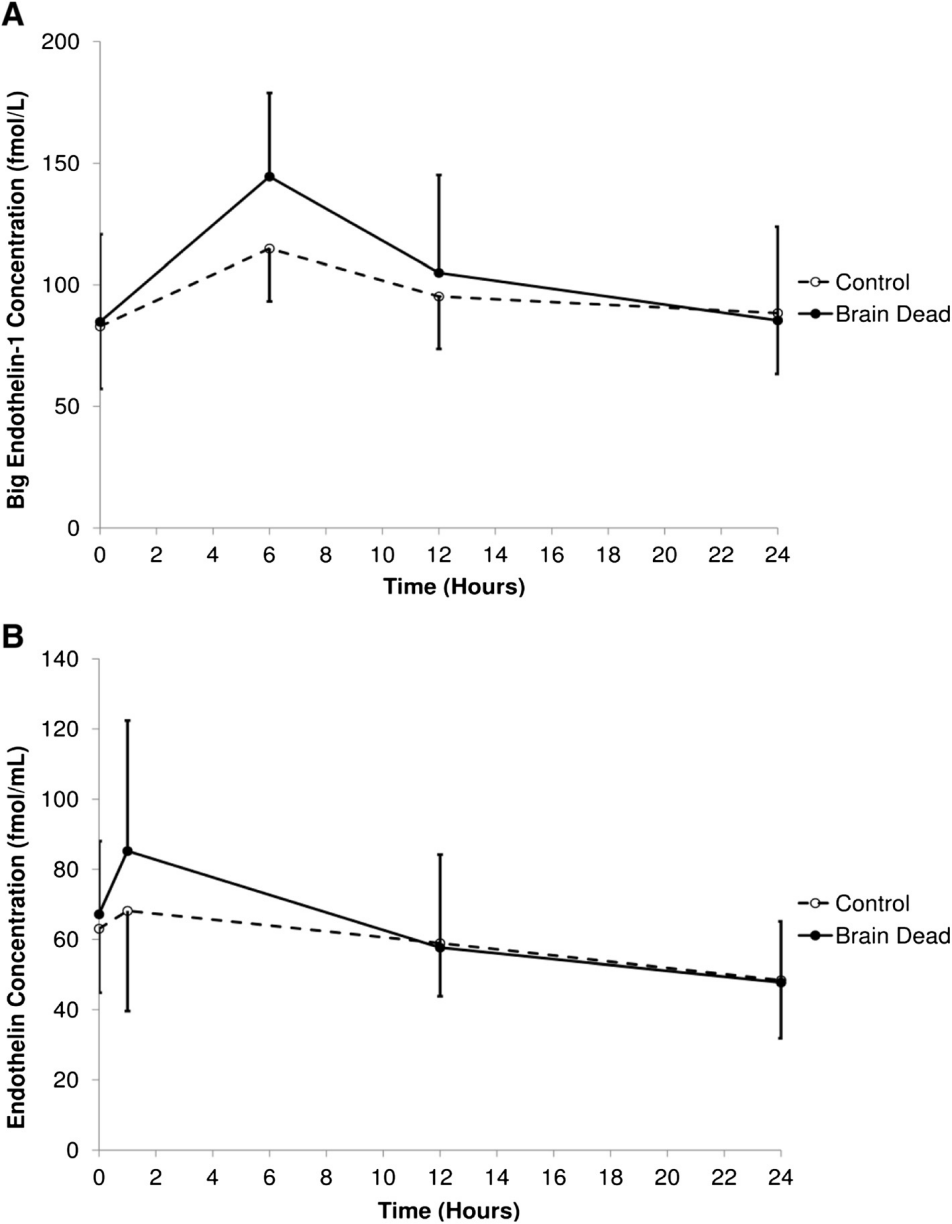
ELISA analysis of the endothelin axis. **a** Big endothelin concentrations. **b** endothelin-1 concentrations. Samples measured in EDTA plasma. Sheep 6 has been excluded from this analysis (brain dead group) due to technical errors in measurement

**Fig. 5:**
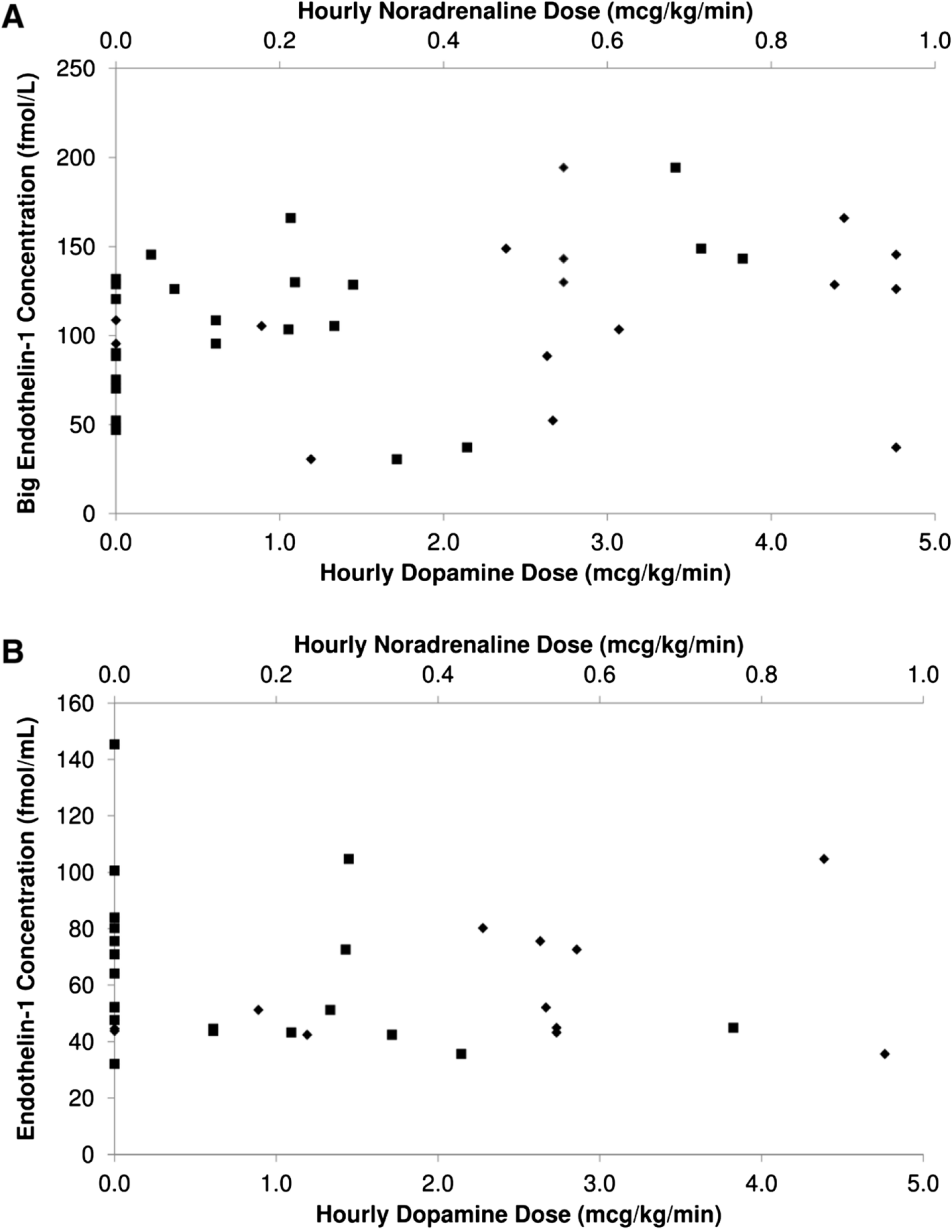
Scatterplot of **a** big endothelin and **b** endothelin-1 concentrations vs average hourly vasoactive infusion doses. *Filled square* = noradrenaline, *filled diamond* = dopamine. Although these agents may stimulate endothelin release, scatterplots do not indicate a correlation between dose and levels measured

Big ET-1 increased in BD sheep 6 h after PST compared to baseline (*p* = 0.002, Fig. 4a). Big ET-1 concentrations also tended to be increased compared to control animals at the same time point (*p* = 0.064). After this early peak, concentrations of big ET-1 returned to baseline at 12 h and remained similar to the non-BD group during the remainder of the protocol (*p* = 0.99 at 24 h). Similarly, ET-1 levels rose by 26.9 % from baseline at 1 h after induction of brain death, approaching statistical significance (*p* = 0.09, Fig. 4b) and then declined to be equivalent to control animals at 12 h. Scatterplots indicated no correlation between hourly average doses of administered vasoactive agents and the observed levels of big ET-1 or ET-1 (Fig. 5).

### Biochemical analysis

**Fig. 6:**
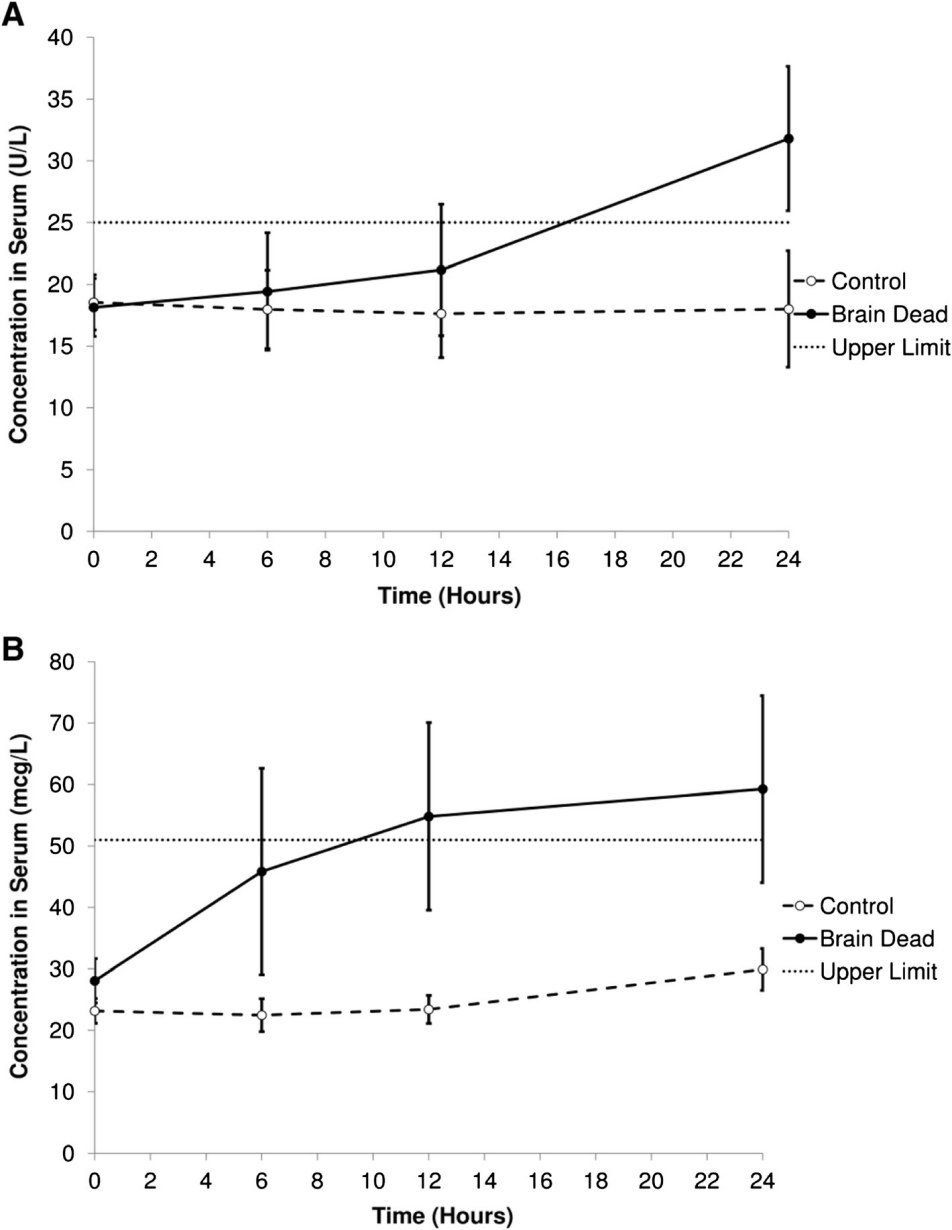
Biochemical results of markers of cardiac injury in brain dead and control animals over 24 h. Upper limit refers to the upper limit of the COBAS reference range. **a** Creatine kinase MB isoenzyme. **b** Myoglobin. Both cardiac markers indicated myocardial injury and necrosis. As expected from their biological properties, myoglobin increased faster, but it did not reach statistical significance. CK-MB increased later in brain dead animals and was statistically significant at 24 h

Circulating myoglobin and CK-MB increased over time in BD animals (Fig. 6), indicating myocardial necrosis; no change was observed in control animals. In BD animals, myoglobin levels increased earlier than CK-MB, however this did not reach statistical significance compared to control animals (*p* = 0.13 at 24 h). CK-MB was significantly elevated in BD compared to control animals at 24 h (*p* = 0.04). Hepatic injury was also evident at 24 h with elevation of both alanine aminotransferase and aspartate aminotransferase in BD animals (*p* < 0.001 for both). Cholestatic enzymes were not elevated, indicating preferential hepatocellular injury. No evidence of renal dysfunction was indicated by elevated creatinine or urea levels (*p* = 0.5 creatinine, *p* = 0.8 urea, BD vs control animals at 24 h).

## Discussion

This is the first report to document a 24-h, clinically relevant, ovine model of brain death and assess systemic and pulmonary endothelin expression. Histological analysis indicated increased inflammation in the BD lung tissue, consistent with previous literature [1, 8]. The components of the endothelin axis were identifiable by immunohistochemical staining, with no demonstrable difference found between groups. This is in contradistinction to previous observations in rodents [8]. ELISA did suggest an early elevation and then resolution of both ET-1 and big ET-1 in plasma after brain death; this may reflect an early release with rapid clearance [33], however no ongoing systemic expression was detected. Observed peak concentrations obtained at 1 and 6 h for ET-1 and big ET-1, respectively, may reflect significant peaks that occurred earlier but were not captured by the sampling time in this study. Oishi et al*.* previously demonstrated that ET-1 peaks as early as 30 min in BD canines, however this elevation was still detectable at 60 min [14]. Another complicating factor in comparing these results to the current study is the nature of sampling; whilst Oishi’s group sampled coronary venous sinus blood (to detect cardiac generated ET-1), our study analysed arterial blood. Systemic levels of ET-1 indicate a spillover effect [34] and therefore may only be detectable in very low concentrations when assessed in this manner due to mixing of blood returning to the pulmonary circulation. Secondly, pulmonary ET_R_B may continue to serve its usual function of clearing circulating ET-1, concealing any detectable elevations in mixed central venous blood [35]. Both of these factors may have contributed to our observed results and provide opportunities for future study.

Data is accumulating of the role of ET-1 in brain death and organ donation; activation of the endothelin axis has been demonstrated early in BD-related pulmonary inflammation [8], it contributes to complications associated with human lung donation [12, 36, 37] and it may contribute to the altered cardiopulmonary haemodynamics observed in the current study, similar to other forms of pulmonary hypertension. As a potent mitogen, ET-1 stimulates smooth muscle hyperplasia and leads to airway remodelling and oedema [11]. Furthermore, ET-1-initiated cell signalling leads to short- and long-term injury, fibrosis and, ultimately, allograft rejection [36]. Thus, endothelin-1 may induce an inflammatory response that continues to manifest itself long after any detectable elevations in plasma concentrations have resolved. Inflammatory cells recruited to the lung by endothelin signalling and increased ET-1 receptors in allograft tissue [8] may be further activated after transplant by ischemia reperfusion injury [11] and the inflammatory state of the recipient [2].

Activation of the sympathetic nervous system during brain death results in dramatic increases in vascular resistance and arterial pressure and contributes to systemic inflammation [38]. Novitzky et al*.* observed that the resulting increase in SVRI and left atrial pressure leads to transfer of up to 72 % of total blood volume to the lower resistance pulmonary vasculature; mPAP and PVRI were also noted to increase with induction of BD [29]. This has been hypothesised to contribute to pulmonary capillary injury after BD. Bittner and colleagues demonstrated that, when observed for 6 h, the PVRI decreased below baseline after the initial sympathetic storm of BD, resulting in increased pulmonary flow and vascular congestion, contributing to increased extravascular lung water content [5]. These authors reported that the decrease in PVRI was secondary to sympathetic failure and increased vascular distensibility [5]. The data over the first 6 h in the present study supports these observations, replicating the early increase in SVRI and MAP, with reduction in PVRI after an early peak. Cardiac index increased from baseline, reaching a maximal value after the SVRI had dropped, resulting in a hyperdynamic circulation. This may maintain the observed reduction in PVRI via distension of pulmonary vessels and pulmonary capillary reserve recruitment [29], in addition to the loss of sympathetic vasoconstriction.

Pulmonary interstitial oedema did not differ between groups. The lack of oedema between the groups may be due to resolution of excess lung water by the end of the protocol. Skilled management likely influenced this outcome; fluid balance was similar between groups, thus preventing iatrogenic pulmonary oedema. The influence of duration of care was posited by Avlonitis et al*.*, who observed that, in a prolonged rat model of brain death (15 min vs 5 h), longer duration of care was associated with better oxygenation and reduced post-transplant PVR despite greater exposure to inflammatory cytokines [3]. This group postulated that the improvement in donor oxygenation at 4 h reflected clearance of neurogenic pulmonary oedema [3]. This finding was replicated in our study, demonstrating impaired oxygenation for the first 2 h, with subsequent recovery to levels similar to controls. Duration of BD donor care also influences recipient survival in humans; time from BD to cold preservation greater than 10 h is associated with a survival advantage at both 5 and 10 years [4]. Hormone resuscitation may have also contributed to the observed effect; methylprednisolone administration reduces extravascular lung water in BD donors [39]. Furthermore, dopamine stimulates alveolar fluid clearance and is another possible mechanism to explain our findings [40].

Whilst the absolute values of mPAP did not greatly exceed the defined cut-off for pulmonary hypertension (>25 mmHg) [41] in the present study, the increased pressure was significantly greater than baseline and does reflect greater resistance that needs to be overcome by a damaged myocardium. The observation that mPAP was elevated at 24 h suggests that the effect of BD on pulmonary pressures may be greater than previously identified. Extended elevations in right ventricular afterload may contribute to the previously identified right ventricular ischaemia and fibrosis, further priming the right ventricle for acute failure in the recipient. Optimisation of ventilation, oxygenation and pH prevented contributions of these factors to the observed increase in pulmonary pressures in the current study.

Administration of catecholamines to the BD animals did represent a difference in care between the two groups. However, this is unlikely to explain the observed findings, because elevated pulmonary pressures remained unchanged when doses of vasopressors were decreased after commencement of hormone therapy. The use of vasoactive agents is common in BD donors [42] and has been demonstrated to reduce inflammation associated with hypotension and resultant poor tissue perfusion [38]. Previous studies of noradrenaline and dopamine infusions in sheep do not support that these agents were causative of the observed alterations in pulmonary pressures [43, 44]. In a study of noradrenaline infusion in healthy and endotoxaemic sheep, Lange et al*.* observed an increase in PVRI in endotoxaemic sheep only [43]. Dopamine infusion in sheep has been associated with an increase in mPAP at rates significantly higher than the doses used in the current study [44, 45]. Although ET-1 can both stimulate [46] and be stimulated by catecholamines [47, 48], comparison of ET-1 and big ET-1 concentrations over time with the average hourly dose of vasoactive agents did not reveal any correlation in the current study and, therefore, does not account for the observed results.

The current study also confirms that BD induces injury in other transplantable organs in sheep. Elevation of hepatic transaminases indicates hepatocellular injury consistent with previous animal models [49, 50]. Serum cholestatic enzyme activities did not increase over time and suggest hepatic injury was not associated with biliary obstruction.

All BD animals required management of haemodynamic collapse with vasopressors. Haemodynamic support was reduced after hormone therapy was initiated. This is in part due to the inclusion of vasopressin but may also reflect improved haemodynamics directly due to hormonal administration. Thyroid hormone may play a role in regulating anaerobic metabolism and cardiovascular stability post-BD, however the benefits of its routine administration remains controversial [15, 51]. The current trial included hormonal resuscitation consistent with local protocols [52].

### Limitations of the study

Several important limitations have been noted in this study. As previously identified, plasma sampling times may have missed very early peaks in ET-1 or big ET-1. More frequent sampling around the induction of BD in future studies will better characterise the time course of ET-1. Small numbers of animals in each group raise the possibility of type 2 error, although pre-clinical animal models have used similar numbers [18, 19]. Myoglobin and CK-MB were chosen to assess for cardiac injury in the current study. Although troponin may reflect cardiac function in the donor, the correlation between troponin levels and recipient outcome remains controversial [53]. Recently published guidelines continue to include CK as a biomarker in assessment of potential heart transplantation donors [54, 55]. Although NT-proBNP has been noted as a potential marker for assessing cardiac function in potential donors [56], it is yet to be included among standard tests for donors [57]. Inflammatory cytokines have been well characterised in other animal models of BD. An ongoing challenge in developing new models is a relative paucity of validated, species-specific analytical methods. Our group continues to develop and validate ovine-specific tests [17, 58], and the presented model will provide a platform to further investigate cytokine expression after BD in future studies.

## Conclusions

The present model replicated the clinical realities leading to delays in organ retrieval upon BD. Haemodynamic disturbances which occur in BD animals have limited the duration of previous studies. By utilising complete haemodynamic monitoring and support in the same fashion as is applied to human donors, it is possible to maintain a BD sheep for 24 h. Whilst sheep undergoing BD demonstrate complex haemodynamic changes similar to those seen in humans, our data also suggests that early haemodynamic and inflammatory derangements may improve over time with aggressive donor management. This reduces the urgency for organ retrieval and supports such timeframes as are frequently encountered in daily clinical practice. However, significant increases in pulmonary blood pressure may be noted up to 24 h after brain death. Big ET-1 and ET-1 are detectable early after BD and may contribute to the inflammatory cascade that primes allografts for post-transplant dysfunction. Endothelin-1 may also be a key factor in the induction of right ventricular dysfunction observed in cardiac transplantation. Further investigation, targeting the endothelin axis, may provide a novel management option in order to improve the condition of transplantable hearts and lungs, increasing the number and quality of allografts available.

## Additional file

Additional file 1:
**Additional file for the investigation of the endothelin axis and associated prolonged cardiopulmonary dysfunction and organ injury in a novel 24-h ovine model of brain death.** Detailed explanation of the protocol used for animal management in development of this novel, clinically relevant, 24-h ovine model of brain death. Detailed description of immunohistochemical staining procedure. Summary of ventilation, blood gas, fluid management and haemodynamic variables over the duration of the study. Summary of biochemical data from the COBAS assessment. Summary of haematoxylin and eosin/immunohistochemical staining results. Representative images of immunohistochemical staining of the endothelin axis. (DOCX 4977 kb)

## Abbreviations

ABG: arterial blood gas
ANOVA: analysis of variance
BD: brain death
Big ET-1: pro-endothelin-1
BIT: brain death induction time
BSA: body surface area
CFAS: calibrator for automated systems
CI: cardiac index
CK-MB: creatine kinase MB fraction
CPP: cerebral perfusion pressure
EDTA: ethylenediaminetetraacetic acid
ET: endothelin
ET_R_A: endothelin A receptor
ET_R_B: endothelin B receptor
F_i_O_2_: fraction of inspired oxygen
ICP: intracranial pressure
LVSWI: left ventricular stroke work index
MAP: mean arterial pressure
MMP: matrix metalloproteinase
mPAP: mean pulmonary artery pressure
P(A-a)O_2_: alveolar-arterial oxygen gradient
PaCO_2_: arterial carbon dioxide partial pressure
PaO_2_: arterial oxygen partial pressure
PAOP: pulmonary arterial occlusion pressure
PST: protocol start time
PVRI: pulmonary vascular resistance index
RVSWI: right ventricular stroke work index
SVI: stroke volume index
SvO_2_: mixed central venous oxygen saturation
SVRI: systemic vascular resistance index
TBS: tris-buffered saline
TBS-T: tris-buffered saline with triton
TIMP: tissue inhibitor of metalloproteinases

## References

[1] Bansal R, Esan A, Hess D, Angel LF, Levine SM, George T, Raoof S (2014). Mechanical ventilatory support in potential lung donor patients. Chest.

[2] Watts RP, Thom O, Fraser JF (2013). Inflammatory signalling associated with brain dead organ donation: from brain injury to brain stem death and posttransplant ischaemia reperfusion injury. J Transplant.

[3] Avlonitis VS, Wigfield CH, Golledge HDR, Kirby JA, Dark JH (2007). Early hemodynamic injury during donor brain death determines the severity of primary graft dysfunction after lung transplantation. Am J Transplant.

[4] Wauters S, Verleden GM, Belmans A, Coosemans W, De Leyn P, Nafteux P, Lerut T, Van Raemdonck D (2011). Donor cause of brain death and related time intervals: does it affect outcome after lung transplantation?. Eur J Cardiothorac Surg.

[5] Bittner HB, Kendall SWH, Chen EP, Craig D, Van Trigt P (1995). The effects of brain death on cardiopulmonary hemodynamics and pulmonary blood flow characteristics. Chest.

[6] OPTN/SRTR 2012 Annual Data Report (2014). Organ Procurement and Transplantation Network (OPTN) and Scientific Registry of Transplant Recipients (SRTR).

[7] Jewell AN, Swamydas M, Castillo CI, Wyan H, Allen LD, McDermott KA, Eddy JM, Dreau D (2010). The endothelin axis stimulates the expression of pro-inflammatory cytokines and pro-migratory molecules in breast cancer. Cancer Invest.

[8] Sutherland A, Ware R, Winterford C, Fraser J (2007). The endothelin axis and gelatinase activity in alveolar macrophages after brain-stem death injury: a pilot Study. J Heart Lung Transplant.

[9] Comellas AP, Briva A (2009). Role of endothelin-1 in acute lung injury. Transl Res.

[10] Kuklin V, Kirov K, Evgenov O, Sovershaev M, Sjoberg J, Kirova S, Bjertnaes L (2004). Novel endothelin receptor antagonist attenuates endotoxin-induced lung injury in sheep. Crit Care Med.

[11] Fagan KA, McMurtry IF, Rodman DM (2001). Role of endothelin-1 in lung disease. Respir Res.

[12] Salama M, Andrukhova O, Hoda MA, Taghavi S, Jaksch P, Heinze G, Klepetko W, Aharinejad S (2010). Concomitant endothelin-1 overexpression in lung transplant donors and recipients predicts primary graft dysfunction. Am J Transplant.

[13] Bittner HB, Kendall SW, Campbell KA, Montine TJ, Van Trigt P (1995). A valid experimental brain death organ donor model. J Heart Lung Transplant.

[14] Oishi Y, Nishimura Y, Tanoue Y, Kajihara N, K-i I, Morita S, Yasui H (2005). Endothelin-1 receptor antagonist prevents deterioration of left ventricular function and coronary flow reserve in brain-dead canine heart. J Heart Lung Transplant.

[15] Ferrera R, Ovize M, Claustrat B, Hadour G (2005). Stable myocardial function and endocrine dysfunction during experimental brain death. J Heart Lung Transplant.

[16] Seok J, Warren HS, Cuenca AG, Mindrinos MN, Baker HV, Xu W, Richards DR, McDonald-Smith GP, Gao H, Hennessy L, Finnerty CC, Lopez CM, Honari S, Moore EE, Minei JP, Cuschieri J, Bankey PE, Johnson JL, Sperry J, Nathens AB, Billiar TR, West MA, Jeschke MG, Klein MB, Gamelli RL, Gibran NS, Brownstein BH, Miller-Graziano C, Calvano SE, Mason PH, Cobb JP, Rahme LG, Lowry SF, Maier RV, Moldawer LL, Herndon DN, Davis RW, Xiao W, Tompkins RG (2013). Genomic responses in mouse models poorly mimic human inflammatory diseases. Proc Natl Acad Sci U S A.

[17] Chemonges S, Tung JP, Fraser JF (2014). Proteogenomics of selective susceptibility to endotoxin using circulating acute phase biomarkers and bioassay development in sheep: a review. Proteome Sci.

[18] Zhai W, Feng R, Huo L, Li J, Zhang S (2009). Mechanism of the protective effects of N-acetylcysteine on the heart of brain-dead Ba-Ma miniature pigs. J Heart Lung Transplant.

[19] Sereinigg M, Stiegler P, Puntschart A, Seifert-Held T, Zmugg G, Wiederstein-Grasser I, Marte W, Marko T, Bradatsch A, Tscheliessnigg K, Stadlbauer-Kollner V (2012). Establishing a brain-death donor model in pigs. Transplant Proc.

[20] Entrican G, Wattegedera SR, Griffiths DJ (2015). Exploiting ovine immunology to improve the relevance of biomedical models. Mol Immunol.

[21] Takao K, Miyakawa T (2014). Genomic responses in mouse models greatly mimic human inflammatory diseases. Proc Natl Acad Sci U S A.

[22] Milani-Nejad N, Janssen PM (2014). Small and large animal models in cardiac contraction research: advantages and disadvantages. Pharmacol Ther.

[23] Scheerlinck J-PY, Snibson KJ, Bowles VM, Sutton P (2008). Biomedical applications of sheep models: from asthma to vaccines. Trends Biotechnol.

[24] Hein WR, Griebel PJ (2003). A road less travelled: large animal models in immunological research. Nat Rev Immunol.

[25] Tung JP, Fraser JF, Wood P, Fung YL (2009). Respiratory burst function of ovine neutrophils. BMC Immunol.

[26] Mariassy AT, Glassberg MK, Salathe M, Maguire F, Wanner A (1996). Endothelial and epithelial sources of endothelin-1 in sheep bronchi. Am J Physiol.

[27] Van der Velden J, Snibson KJ (2011). Airway disease: the use of large animal models for drug discovery. Pulm Pharmacol Ther.

[28] Kumar S, Oishi PE, Rafikov R, Aggarwal S, Hou Y, Datar SA, Sharma S, Azakie A, Fineman JR, Black SM (2013). Tezosentan increases nitric oxide signaling via enhanced hydrogen peroxide generation in lambs with surgically induced acute increases in pulmonary blood flow. J Cell Biochem.

[29] Novitzky D, Wicomb WN, Rose AG, Cooper DK, Reichart B (1987). Pathophysiology of pulmonary edema following experimental brain death in the chacma baboon. Ann Thorac Surg.

[30] ANZDATA Registry Report 2012 (2013). ANZDATA Registry.

[31] Nataatmadja M, Passmore M, Russell FD, Prabowo S, Corley A, Fraser JF (2014). Angiotensin receptors as sensitive markers of acute bronchiole injury after lung transplantation. Lung.

[32] Diggle P, Heagerty P, Liang K-L, Zeger SL (2002). Analysis of longitudinal data.

[33] Parker JD, Thiessen JJ, Reilly R, Tong JH, Stewart DJ, Pandey AS (1999). Human endothelin-1 clearance kinetics revealed by a radiotracer technique. J Pharmacol Exp Ther.

[34] Wagner OF, Christ G, Wojta J, Vierhapper H, Parzer S, Nowotny PJ, Schneider B, Waldhäusl W, Binder BR (1992). Polar secretion of endothelin-1 by cultured endothelial cells. J Biol Chem.

[35] Dupuis J, Goresky CA, Fournier A (1996). Pulmonary clearance of circulating endothelin-1 in dogs in vivo: exclusive role of ETB receptors. J Appl Physiol.

[36] Salama M, Andrukhova O, Jaksch P, Taghavi S, Kelpetko W, Dekan G, Aharinejad S (2011). Endothelin-1 governs proliferation and migration of bronchoalveolar lavage-derived lung mesenchymal stem cells in bronchiolitis obliterans syndrome. Transplantation.

[37] Salama M, Jaksch P, Andrukhova O, Taghavi S, Klepetko W, Aharinejad S (2010). Endothelin-1 is a useful biomarker for early detection of bronchiolitis obliterans in lung transplant recipients. J Thorac Cardiovasc Surg.

[38] Avlonitis VS, Wigfield CH, Kirby JA, Dark JH (2005). The hemodynamic mechanisms of lung injury and systemic inflammatory response following brain death in the transplant donor. Am J Transplant.

[39] Venkateswaran RV, Patchell VB, Wilson IC, Mascaro JG, Thompson RD, Quinn DW, Stockley RA, Coote JH, Bonser RS (2008). Early donor management increases the retrieval rate of lungs for transplantation. Ann Thorac Surg.

[40] Ware LB, Fang X, Wang Y, Sakuma T, Hall TS (1985). Matthay MA (2002) Selected contribution: mechanisms that may stimulate the resolution of alveolar edema in the transplanted human lung. J Appl Physiol.

[41] Chemla D, Castelain V, Herve P, Lecarpentier Y, Brimioulle S (2002). Haemodynamic evaluation of pulmonary hypertension. Eur Respir J.

[42] Dare A, Bartlett A, Fraser J (2012). Critical care of the potential organ donor. Curr Neurol Neurosci Rep.

[43] Lange M, Broking K, Hucklenbruch C, Ertmer C, Van Aken H, Lucke M, Bone HG, Westphal M (2007). Hemodynamic effects of titrated norepinephrine in healthy versus endotoxemic sheep. J Endotoxin Res.

[44] Feltes TF, Hansen TN, Martin CG, Leblanc AL, Smith S, Giesler ME (1987). The effects of dopamine infusion on regional blood flow in newborn lambs. Pediatr Res.

[45] Teboul JL, Douguet D, Mercat A, Depret J, Richard C, Zelter M (1998). Effects of catecholamines on the pulmonary venous bed in sheep. Crit Care Med.

[46] Boarder MR, Marriott DB (1991). Endothelin-1 stimulation of noradrenaline and adrenaline release from adrenal chromaffin cells. Biochem Pharmacol.

[47] Morimoto T, Hasegawa K, Wada H, Kakita T, Kaburagi S, Yanazume T, Sasayama S (2001). Calcineurin-GATA4 pathway is involved in beta-adrenergic agonist-responsive endothelin-1 transcription in cardiac myocytes. J Biol Chem.

[48] Yanagisawa M, Kurihara H, Kimura S, Tomobe Y, Kobayashi M, Mitsui Y, Yazaki Y, Goto K, Masaki T (1988). A novel potent vasoconstrictor peptide produced by vascular endothelial cells. Nature.

[49] Hoti E, Levesque E, Sebagh M, Heneghan HM, Khalfallah M, Castaing D, Azoulay D (2014). Liver transplantation with grafts from donors who die from suicide by hanging: a matched cohort study. Transplantation.

[50] Zhu C, Li J, Zhang G, Zhang Y, Zhai W, Shi J, Li Z, Zhang S (2010). Brain death disrupts structure and function of pig liver. Transplant Proc.

[51] Macdonald PS, Aneman A, Bhonagiri D, Jones D, O'Callaghan G, Silvester W, Watson A, Dobb G (2012). A systematic review and meta-analysis of clinical trials of thyroid hormone administration to brain dead potential organ donors. Crit Care Med.

[52] National Guidelines for Organ and Tissue Donation 4th Edition (2008). Australasian transplant coordinators association incorporated, organ and tissue authority Australia.

[53] Dronavalli VB, Banner NR, Bonser RS (2010). Assessment of the potential heart donor: a role for biomarkers?. J Am Coll Cardiol.

[54] Kilic A, Emani S, Sai-Sudhakar CB, Higgins RSD, Whitson BA (2014). Donor selection in heart transplantation. J Thoracic Dis.

[55] Organ Procurement and Transplantation Network (2015) Policy 2: Deceased Donor Organ Procurement. http://optn.transplant.hrsa.gov/governance/policies/. Accessed 3 August 2015

[56] Dronavalli VB, Ranasinghe AM, Venkateswaran RJ, James SR, McCabe CJ, Wilson IC, Mascaro JG, Bonser RS (2010). N-terminal pro-brain-type natriuretic peptide: a biochemical surrogate of cardiac function in the potential heart donor. Eur J Cardiothorac Surg.

[57] Costanzo MR, Dipchand A, Starling R, Anderson A, Chan M, Desai S, Fedson S, Fisher P, Gonzales-Stawinski G, Martinelli L, McGiffin D, Smith J, Taylor D, Meiser B, Webber S, Baran D, Carboni M, Dengler T, Feldman D, Frigerio M, Kfoury A, Kim D, Kobashigawa J, Shullo M, Stehlik J, Teuteberg J, Uber P, Zuckermann A, Hunt S, Burch M, Bhat G, Canter C, Chinnock R, Crespo-Leiro M, Delgado R, Dobbels F, Grady K, Kao W, Lamour J, Parry G, Patel J, Pini D, Towbin J, Wolfel G, Delgado D, Eisen H, Goldberg L, Hosenpud J, Johnson M, Keogh A, Lewis C, O'Connell J, Rogers J, Ross H, Russell S, Vanhaecke J (2010). The International Society of Heart and Lung Transplantation Guidelines for the care of heart transplant recipients. J Heart Lung Transplant.

[58] Passmore M, Nataatmadja M, Fraser JF, Passmore M, Nataatmadja M, Fraser JF (2009). Selection of reference genes for normalisation of real-time RT-PCR in brain-stem death injury in Ovis aries. BMC Mol Biol.

