# Supplementary material for: Novel 24-h ovine model of brain death to study the profile of the endothelin axis during cardiopulmonary injury
Source: Intensive Care Med Exp. 2015 Nov 24;3:31. doi: 10.1186/s40635-015-0067-9 (PMC4656265; doi:10.1186/s40635-015-0067-9)
Supplement: Additional file 1: — Additional file for the investigation of the endothelin axis and associated prolonged cardiopulmonary dysfunction and organ injury in a novel 24-h ovine model of brain death. Detailed explanation of the protocol used for animal management in development of this novel, clinically relevant, 24-h ovine model of brain death. Detailed description of immunohistochemical staining procedure. Summary of ventilation, blood gas, fluid management and haemodynamic variables over the duration of the study. Summary of biochemical data from the COBAS assessment. Summary of haematoxylin and eosin/immunohistochemical staining results. Representative images of immunohistochemical staining of the endothelin axis. (DOCX 4977 kb) [file 40635_2015_67_MOESM1_ESM.docx]

*Additional File for the Novel 24 hour ovine model of brain death to study the profile of the endothelin axis during cardiopulmonary injury*

Contents

[Animal Management 2](#_Toc434157497)

[Anaesthesia Induction, Surgical Preparation and Monitoring Setup 2](#_Toc434157498)

[Induction of Brain Death 3](#_Toc434157499)

[Protocol for Anaesthesia, Sedation and Analgesia in Control Animals after Surgical Preparation 4](#_Toc434157500)

[Protocol for Anaesthesia, Sedation and Analgesia in Brain Dead Animals after Surgical Preparation 4](#_Toc434157501)

[Protocol for Haemodynamic, Ventilatory and Electrolyte Management in all Animals 4](#_Toc434157502)

[Protocol for Metabolic Management, Hormone Resuscitation and Animal Sacrifice in all Animals 5](#_Toc434157503)

[References: 6](#_Toc434157504)

[Immunohistochemistry Staining Procedure 7](#_Toc434157505)

[Summary of Animal Data 8](#_Toc434157506)

[Ventilatory, Arterial Blood Gas and Fluid Balance Data 8](#_Toc434157507)

[Summary of Systemic Haemodynamic Variables 9](#_Toc434157508)

[Summary of Pulmonary Haemodynamic Variables 10](#_Toc434157509)

[Summary of Biochemical Data 11](#_Toc434157510)

[Summary of Haematoxylin and Eosin and Immunohistochemical Staining 12](#_Toc434157511)

[Representative Images of Immunohistochemical Staining of the Endothelin Axis. 13](#_Toc434157512)

# Animal Management

## Anaesthesia Induction, Surgical Preparation and Monitoring Setup

Animals were divided into two groups (brain dead vs control) of six sheep each, for a total of 12 animals. Initial surgical preparation was identical in both groups of animals. After overnight fasting, animals were administered local anaesthesia (lignocaine 1%) and the right external jugular vein was cannulated using a 7 Fr triple lumen central venous catheter (Arrow-Howes, Research Triangle Park, North Carolina, USA). The left external jugular vein was accessed via an 8 Fr sheath for later placement of a pulmonary artery catheter (Edwards Lifesciences, Irvine, California, USA). Buprenorphine, cefalotin and gentamicin were then administered. Table 1 in the main manuscript lists details of medications used in this study. General anaesthesia was induced with midazolam and alfaxalone, and animals were intubated under direct laryngoscopy. Mechanical ventilation was commenced using a Galileo ventilator (Hamilton Medical, Reno, Nevada, USA) with continuous quantitative capnography. Initial ventilator settings were Synchronised Controlled Mandatory Ventilation, F_i_O_2_ 0.5, tidal volume 10 mL/kg, 20 breaths per minute and positive end-expiratory pressure (PEEP) of 5 cmH_2_O. Ventilation was adjusted to keep ETCO­­_2_ at 35-40 mmHg, with mild hyperventilation to reduce animal discomfort and allow invasive ventilation of sheep in the awake state.(1,2)

Anaesthesia was maintained during surgical preparation with continuous infusions of alfaxalone and ketamine and adjusted to maintain a surgical plane (monitored by observing heart rate, blood pressure, respiratory efforts and rate, eyelash reflexes, chewing and jaw movements). Midazolam infusion was commenced if alfaxalone exceeded 250 mg/hr.

Continuous electrocardiography and oxygen saturation monitoring was commenced directly after intubation. Invasive arterial blood pressure monitoring was performed after cannulation of the right facial artery under direct surgical visualisation. Physiologic variables were monitored using a Marquette Solar 8000 monitor (GE Healthcare, Little Chalfont, UK) and recorded every five seconds with custom software. A 7.5 Fr pulmonary artery catheter was inserted via the previously placed sheath and continuous monitoring of the mixed venous oxygen saturation (SvO_2_), cardiac output and body temperature were commenced using a Vigilance II Monitor (Edwards Lifesciences, Irvine, California, USA). Data were recorded every two seconds using manufacturer provided software. Cardiac index, systemic vascular resistance index (SVRI), pulmonary vascular resistance index (PVRI), stroke volume index and right ventricular and left ventricular stroke work index were calculated according to standard equations.(3)

Pulmonary artery diastolic pressure was used to calculate PVRI in lieu of repeated measurements of pulmonary artery occlusion pressure (PAOP) to minimise the risk of iatrogenic injury. The PAOP was measured every six hours to ensure consistency with the pulmonary diastolic pressure. Calculation of the body surface area (BSA) was performed using the equation, BSA=0.094x(weight)^0.67^.(4) A size 10 Portex tracheostomy tube (Smiths Medical, London, UK) was surgically placed and the endotracheal tube removed. A transurethral urinary catheter was inserted to monitor urine output.

Finally, intracranial access was obtained in all animals through a surgical incision midway between the midline and lateral edge of the cranium, rostral to the animal's horn base. A burr hole was created to reveal the dura and an intracranial pressure monitor was placed. This point was designated the protocol start time in control animals.

## Induction of Brain Death

In animals allocated to brain death, a second burr hole was created on the opposite side of the head. A 16 Fr Foley catheter was then inserted extradurally. Brain death was induced via inflation of the catheter balloon with 20 mL saline in 5 mL aliquots. One milliliter aliquots were then used to ensure that the cerebral perfusion pressure (CPP, defined as the mean arterial pressure less the intracranial pressure) remained negative for 30 minutes,(5) preventing cerebral blood flow, and that no additional sympathetic response or seizure activity occurred in response to the boluses. Confirmation of brain death was achieved by continuously negative CPP for greater than 30 minutes, loss of pupillary and corneal reflexes and lack of respiratory efforts. This was designated as the protocol start time for BD animals.

## Protocol for Anaesthesia, Sedation and Analgesia in Control Animals after Surgical Preparation

After the protocol start time in control animals, six hourly buprenorphine was continued for analgesia, while anaesthesia remained according to the surgical plane. Anaesthesia was ceased at twelve hours after the protocol start time. Ketamine, alfaxalone and midazolam were able to be administered to non-BD animals after this time to prevent distress and ensure animal well-being. If required, these were titrated to clinical sedation, whereby the animals were calm but easily rousable. Muscle relaxants were not used at any time to allow monitoring for signs of animal distress.

## Protocol for Anaesthesia, Sedation and Analgesia in Brain Dead Animals after Surgical Preparation

Anaesthetic and analgesic agents were continued after brain death to maintain consistency of care between groups. Ketamine and alfaxalone were reduced to 2 mg/hr and 1 mg/hr respectively after diagnosis of brain death, reflecting adjustment to maintain a ‘surgical plane’ in these animals. Midazolam was ceased if it had been used. Six hourly buprenorphine was also continued. Ketamine and alfaxalone were ceased at 12 hours in BD animals.

## Protocol for Haemodynamic, Ventilatory and Electrolyte Management in all Animals

Hartmann’s solution was initially administered at 2 mL/kg/hr and titrated to maintain a right atrial pressure of 8–12 mmHg. Further fluid boluses of crystalloid could be administered to maintain haemodynamics and urine output greater than 0.5 mL/kg/hr. Hypotension, defined as systolic blood pressure of <90 mmHg or MAP <60 mmHg, was treated with intravascular fluid optimisation and then dopamine or noradrenaline could be commenced.

Arterial blood gases (ABG) were measured with a Radiometer ABL-825 analyser (Copenhagen, Denmark) every two hours or as required. Ventilatory adjustments were made to achieve P_a_O_2_ of greater than 100 mmHg (13.3 kPa) with a minimum F_i_O_2_ of 0.3, and P_a_CO_2_ of 35 mmHg (4.6 kPa) and normal pH. Potassium and calcium were replaced as directed by ABG results.

## Protocol for Metabolic Management, Hormone Resuscitation and Animal Sacrifice in all Animals

Blood glucose levels were maintained between 6 and 10 mmol/L using insulin or dextrose infusions as necessary. Body temperature was manipulated with warmed fluids, warming blankets, heat lamps and manipulation of the operating theatre environmental temperature to maintain normothermia. Diabetes insipidus was defined as urine output greater than 300 mL/hr for two consecutive hours.(6) This was treated with desmopressin and adequate fluid replacement.

Hormone resuscitation therapy with vasopressin, methylprednisolone and liothyronine was commenced in all animals at 12 hours after the designated protocol start time.(7) Animals were euthanised at 24 hours with sodium pentobarbitone.

### References:

1. Maybauer MO, Maybauer DM, Fraser JF, Traber LD, Westphal M, Cox RA, Huda R, Nakano YY, Enkhbaatar P, Hawkins HK, Herndon DN, Traber DL (2007) **Ceftazidime improves hemodynamics and oxygenation in ovine smoke inhalation injury and septic shock**. [Erratum appears in Intensive Care Med. 2007 Jul;33(7):1228]. Intensive Care Med 33 (7):1219-1227

2. Maybauer DM, Maybauer MO, Traber LD, Westphal M, Nakano YY, Enkhbaatar P, Morita N, Herndon DN, Traber DL (2006) **Effects of severe smoke inhalation injury and septic shock on global hemodynamics and microvascular blood flow in sheep**. Shock 26 (5):489-495.

3. McGee W, Headly J, Frazier J (eds.): **Quick Guide to Cardiopulmonary Care**, 2nd edn: Edwards Lifesciences; 2012.

4. Bennett J: **Regional Body Surface Area of Sheep**. *Journal of Agricultural Science* 1973, 81:429-432.

5. Novitzky D, Wicomb WN, Rose AG, Cooper DK, Reichart B (1987) **Pathophysiology of pulmonary edema following experimental brain death in the chacma baboon.** Ann Thorac Surg 43 (3):288-294

6. Dumont AS, Nemergut EC, 2nd, Jane JA, Jr., Laws ER, Jr.: **Postoperative care following pituitary surgery**. *J Intensive Care Med* 2005, 20(3):127-140.

7. **National Guidelines for Organ and Tissue Donation** 4th Edition (2008). 4th edn. Australasian Transplant Coordinators Association Incorporated, Organ and Tissue Authority Australia

# Immunohistochemistry Staining Procedure

Primary antibodies used for immunohistochemistry included monoclonal anti-endothelin 1 (ET-1, Sigma Aldrich, St Louis, Mo), polyclonal anti-endothelin A receptor (ET_R_A), anti-endothelin B receptor (ET_R_B) and anti-matrix-metalloproteinase (MMP) -2 (Merck Millipore, Billerica, MA), polyclonal anti-MMP-9 (Biorbyt, San Francisco, CA) and polyclonal anti-tissue inhibitor of metalloproteinase (TIMP)-1 and anti-TIMP-2 (Bioss, Woburn, MA). After trials for optimisation of concentration, the antibodies were diluted in TBS 0.5% Triton-100 (TBS-T) in the following ratios; ET-1 1:100, ET_R_A 1:20, ET_R_B 1:100, MMP-2 1:100, MMP-9 1:50, TIMP-1 1:50 and TIMP-2 1:50.

After sections were rehydrated, they were washed in Tris buffered saline (TBS) and epitope heat retrieval was performed using citrate buffer solution (10mM Sodium Citrate, 0.05% Tween-20, pH 6.0) for 20 minutes. After cooling, the slides were rinsed in distilled water and endogenous peroxidase activity was quenched with 3% hydrogen peroxide solution for 5 minutes. Slides were washed and then placed in TBS prior to application of 1:10 normal horse serum in TBS-T for one hour in a humidified chamber at room temperature. Diluted primary antibodies were applied and slides were then incubated at 4°C (1 hour for ET-1, ET_R_A, ET_R_B and MMP-2 and 24 hours for MMP-9, TIMP-1 and TIMP-2).

Following incubation, slides were rinsed with TBS-T and a biotinylated secondary antibody was applied (Vectastain ABC system, Vector Labs, Burlingame, CA) and incubated in a humidified chamber at room temperature for 60 minutes. After rinsing with TBS-T, an avidin/biotin complex was applied and the slides were again incubated in a humidified chamber at room temperature for 60 minutes. Slides were rinsed in TBS-T and then developed with 3,3’-diaminobenzidine to reveal brown staining peroxidase activity. After staining with Mayer’s haematoxylin, samples were dehydrated and mounted with DePx (BDH Laboratories, Poole, England). Negative controls were created by completing the same steps with exclusion of the primary antibodies.

# Summary of Animal Data

## Ventilatory, Arterial Blood Gas and Fluid Balance Data

|  |  | | **Control** | | | | **Brain Dead** |
| --- | --- | --- | --- | --- | --- | --- | --- |
| **Ventilation at 24 Hours** | |  | |  | | | |
| *Minute Ventilation* | *(L/Min)* | | 8.2 ± 1.2 | | | 9.8 ± 1.3 | |
| *Respiratory Rate* | *(Breaths/Min)* | | 19.6 ± 2.6 | | | 22.1 ± 2.7 | |
| *Tidal Volume* | *(Litres)* | | 428.1 ± 42.5 | | | 456.9 ± 44.7 | |
| *Positive End Expiratory Pressure* | *(cmH_2_O)* | | 6.8 ± 1.2 | | | 7.5 ± 1.3 | |
| *Plateau Pressure* | *(cmH_2_O)* | | 18.9 ± 1.8 | | | 19.3 ± 2.0 | |
| *Static Compliance* | *(L/cmH_2_O)* | | 35.9 ± 5.5 | | | 39.2 ± 5.8 | |
| **Blood Gas Results at 24 hours** | |  | | |  | | |
| *P_(A-a)_O_2_* | *(mmHg)* | | 23.4 ± 25.5 | | | 14.6 ± 26.7 | |
| *P_a_O_2_:F_i_O_2_* |  | | 442.9 ± 44.7 | | | 447.6 ± 46.9 | |
| *PaCO_2_* | *(mmHg)* | | 27 ± 4.5 | | | 31.9 ± 4.7 | |
| *pH* |  | | 7.46 ± 0.06 | | | 7.38 ± 0.09 | |
| *Lactate* | *(Mol/L)* | | 1.1 ± 0.5† | | | 1.9 ± 0.6 | |
| **Fluid Parameters at 24 hours** | |  | | |  | | |
| *Cumulative Fluid Administered* | *L* | | 4.7 ± 1.7† | | | 15.2 ± 12.5 | |
| *Cumulative Urine Output* | *L* | | 2.6 ± 0.9† | | | 12.8 ± 11.6 | |
| *Fluid Balance* | *L* | | 2.1 ± 0.8 | | | 2.4 ± 1.7 | |

† - p < 0.05 CP vs BDP ˜ - p < 0.05 vs baseline

*Maximum/Minimum values given occurred within 15 minutes*

## Summary of Systemic Haemodynamic Variables

**Control** **Brain Dead**

| **Systemic Haemodynamics** | |  | |  | |
| --- | --- | --- | --- | --- | --- |
| *Heart Rate (beats/min)* | *Baseline* | | 122 ± 1 | | 101 ± 5 |
|  | *Maximum* | | 125 ± 30† | | 220 ± 73˜ |
|  | *Average First Hour* | | 124 ± 1 † | | 174 ± 15˜ |
|  | *Average at 24 Hours* | | 105 ± 8 | | 100 ± 9 |
| *Cardiac Index (L/min)* | *Baseline* | | 4.0 ± 0.15 | | 4.5 ± 0.18 |
|  | *Maximum* | | 4.7 ± 1.2† | | 7.5 ± 2.19˜ |
|  | *Average at 24 Hours* | | 4.6 ± 0.5 | | 4.6 ± 0.6 |
| *Mean Arterial Pressure (mmHg)* | *Baseline* | | 107 ± 2 | | 99 ± 3 |
|  | *Maximum* | | 110 ± 18† | | 190 ± 40˜ |
|  | *Average at 24 Hours* | | 122 ± 5† | | 83 ± 5˜ |
| *Central Venous Pressure (mmHg)* | *Baseline* | | 7.5 ± 3.2 | | 6.2 ± 5.0 |
|  | *Maximum* | | 8.5 ± 4.3 | | 11.3 ± 5.3 |
|  | *Average at 24 Hours* | | 9.7 ± 1.8 | | 10.9 ± 1.7 |
| *SvO_2_ (%)* | *Baseline* | | 78.7 ± 9.5† | | 67 ± 11.8 |
|  | *Minimum* | | 77.2 ± 13.1 | | 67.5 ± 6.3 |
|  | *Average at 24 Hours* | | 78.2 ± 4.2 | | 75.5 ± 5.0 |
| *Systemic Vascular Resistance Index (dyn.s.cm^-5^)* | *Baseline* | | 1808 ± 49 | | 1741 ± 60 |
|  | *Maximum* | | 1885 ± 401† | | 3718 ± 77˜ |
|  | *Average at 24 Hours* | | 1973 ± 202† | | 1317 ± 212 |
| *Left Ventricular Stroke Work Index (g.m/m^2^/beat)* | *Baseline* | | 47 ± 1.7 | | 53 ± 1.7 |
|  | *Maximum* | | 49 ± 15.9 | | 57 ± 23.4˜ |
|  | *Average at 24 Hours* | | 65 ± 5.8†˜ | | 45 ± 6.1˜ |

† - p < 0.05 CP vs BDP ˜ - p < 0.05 vs baseline

*Maximum/Minimum values given occurred within 15 minutes*

## Summary of Pulmonary Haemodynamic Variables

**Control** **Brain Dead**

| **Pulmonary Haemodynamics** | |  | |  | |
| --- | --- | --- | --- | --- | --- |
| *Mean Pulmonary Artery Pressure (mmHg)* | *Baseline* | | 19 ± 0.8 | | 16 ± 0.2 |
|  | *Maximum* | | 20 ± 4.9† | | 30 ± 13˜ |
|  | *Average at 24 Hours* | | 21 ± 2.2 | | 25 ± 2.2˜ |
| *Pulmonary Vascular Resistance Index (dyn.s.cm^-5^)* | *Baseline* | | 54 ± 3.9 | | 50 ± 3.1 |
|  | *Maximum* | | 57 ± 16.9† | | 123 ± 77˜ |
|  | *Average at 24 Hours* | | 70 ± 19 | | 61 ± 19 |
| *Right Ventricular Stroke Work Index (g.m/m^2^/beat)* | *Baseline* | | 6 ± 0.4 | | 7 ± 0.2 |
|  | *Maximum* | | 6 ± 0.4 | | 7 ± 2.6 |
|  | *Average at 24 Hours* | | 8 ± 1.0 | | 9 ± 1.0˜ |

† - p < 0.05 CP vs BDP ˜ - p < 0.05 vs baseline

*Maximum/Minimum values given occurred within 15 minutes*

# Summary of Biochemical Data

|  | | | **Control** | | **Brain Dead** |
| --- | --- | --- | --- | --- | --- |
| **Cardiac Markers** | | |  |  | |
| *Myoglobin* | *mcg/L* | *Baseline* | 23.2 ± 2.0 | | 28.7 ± 3.6 |
|  |  | *24 Hours* | 29.9 ± 3.4 | | 59.3 ± 15 |
| *Creatine Kinase MB* | *U/L* | *Baseline* | 18.5 ± 2.2 | | 18.1 ± 2.3 |
|  |  | *24 Hours* | 18 ± 4.7 | | 31.8 ± 5.8 |
| **Hepatic Markers** | | |  |  | |
| *Total Bilirubin* | *μmol/L* | *Baseline* | 1.27 ± 0.4 | | 0.9 ± 0.5 |
|  |  | *24 Hours* | 4.0 ± 3.8†~ | | 0.8 ± 0.6 |
| *Conjugated Bilirubin* | *μmol/L* | *Baseline* | 1.3 ± 0.6 | | 1.0 ± 0.6 |
|  |  | *24 Hours* | 3.2 ± 1.8†~ | | 1.0 ± 0.7 |
| *Alkaline Phosphatase* | *U/L* | *Baseline* | 105 .2 ± 29.3 | | 84.1 ± 23.7 |
|  |  | *24 Hours* | 76.2 ± 19.4 | | 84.6 ± 25.5 |
| γ*-Glutamyl Transferase* | *U/L* | *Baseline* | 58.7 ± 6.8 | | 56.8 ± 2.6 |
|  |  | *24 Hours* | 55.0 ± 6.4 | | 50.3 ± 4.0 |
| *Alanine Aminotransferase* | *U/L* | *Baseline* | 9.8 ± 1.2 | | 8.0 ± 1.1 |
|  |  | *24 Hours* | 16.4 ± 1.1† | | 75.5 ± 39.5 |
| *Aspartate Aminotransferase* | *U/L* | *Baseline* | 78.6 ± 4.7 | | 79.9 ± 7.2 |
|  |  | *24 Hours* | 139.3 ± 9.8† | | 596.0 ± 291.1 |
| *Albumin* | *g/L* | *Baseline* | 30 ± 2.4 | | 31 ± 4.6 |
|  |  | *24 Hours* | 29 ± 0.8 | | 24 ± 4.4~ |
| *Total Protein* | *g/L* | *Baseline* | 61 ± 3.9 | | 66 ± 8.6 |
|  |  | *24 Hours* | 60 ± 5.6 | | 51 ± 7.3~ |
| *Lactate Dehydrogenase* | *U/L* | *Baseline* | 470 ± 90 | | 402 ± 83 |
|  |  | *24 Hours* | 791 ± 254† | | 2569 ± 2977~ |
| **Renal Markers** | | |  |  | |
| *Urea* | *mmol/L* | *Baseline* | 7.7 ± 0.8 | | 6.5 ± 0.7 |
|  |  | *24 Hours* | 4.3 ± 0.2 | | 2.5 ± 0.5 |
| *Creatinine* | *μmol/L* | *Baseline* | 68.4 ± 2.6 | | 71.3 ± 7.9 |
|  |  | *24 Hours* | 67.2 ± 3.5 | | 57.1 ± 6.4 |
| *Phosphate* | *mmol/L* | *Baseline* | 2.3 ± 0.6 | | 1.9 ± 0.4 |
|  |  | *24 Hours* | 1.2 ± 0.3~ | | 1.5 ± 0.7 |

† - p < 0.05 CP vs BDP ˜ - p < 0.05 vs baseline

# Summary of Haematoxylin and Eosin and Immunohistochemical Staining

| **Sheep** | **HE Score** | **Neutrophils (/Field)*** | **ET-1** | **ET_R_A** | **ET_R_B** | **MMP-2** | **MMP-9** | **TIMP-1** | **TIMP-2** |
| --- | --- | --- | --- | --- | --- | --- | --- | --- | --- |
| **Brain Dead** | | | | | | | | | |
| 1 | ++ | 19 | + | 0 | + | 0 | 0 | + | ++ |
| 2 | ++ | 10 | + | 0 | + | + | + | + | + |
| 4 | + | 3 | + | 0 | + | 0 | 0 | 0 | + |
| 6 | ++ | 11 | 0 | 0 | + | 0 | 0 | + | + |
| 8 | ++ | 6 | 0 | 0 | + | + | 0 | 0 | 0 |
| 10 | + | 4 | 0 | 0 | + | + | 0 | 0 | + |
| **Control** | | | | | | | | | |
| 3 | 0 | 4 | 0 | 0 | + | + | + | 0 | 0 |
| 5 | 0 | 5 | + | 0 | + | 0 | 0 | 0 | + |
| 7 | + | 9 | + | + | + | + | 0 | 0 | 0 |
| 9 | ++ | 3 | 0 | 0 | + | 0 | 0 | 0 | 0 |
| 11 | ++ | 6 | + | + | + | 0 | + | 0 | + |
| 12 | 0 | 4 | 0 | 0 | + | 0 | 0 | + | 0 |
| *P-Value* | 0.014 | 0.018 | 1.0 | 1.0 | 1.0 | 1.0 | 1.0 | 1.0 | 1.0 |

** Neutrophils counted by microscopy in 5 fields under 63x power and then averaged.*

# Representative Images of Immunohistochemical Staining of the Endothelin Axis.

| ***A)***  **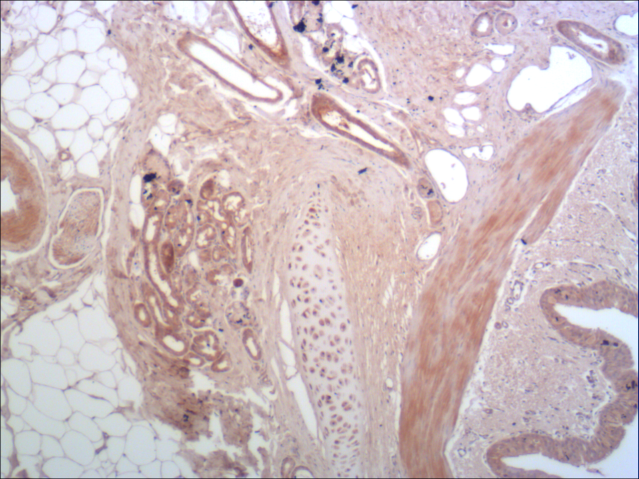** | **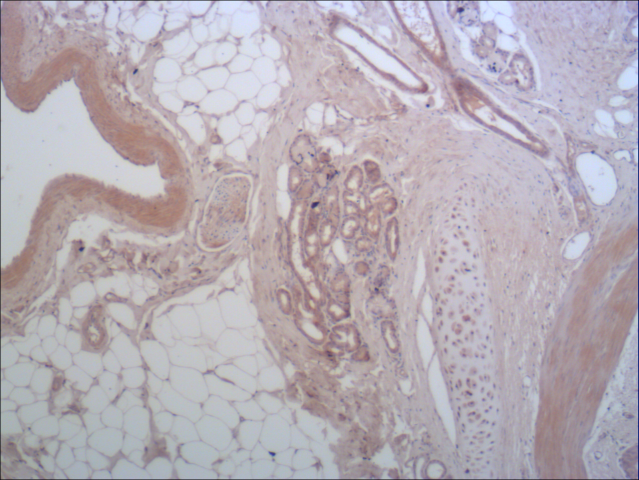** |
| --- | --- |
| ***B)***  *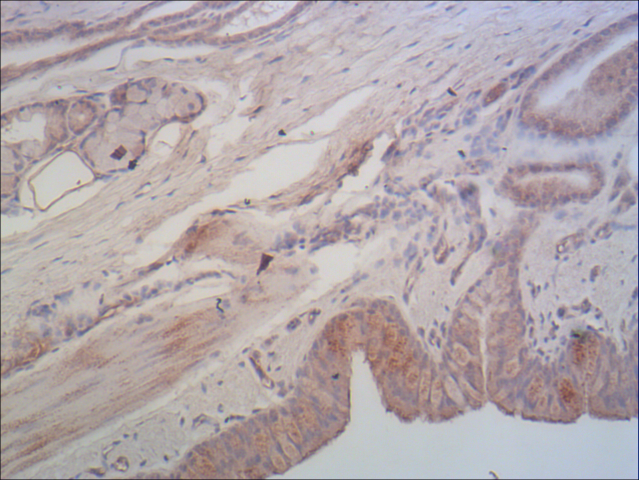* | **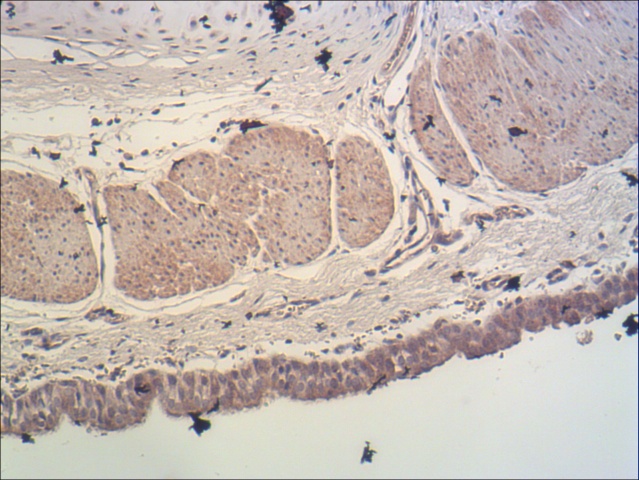** |
| ***C)***  *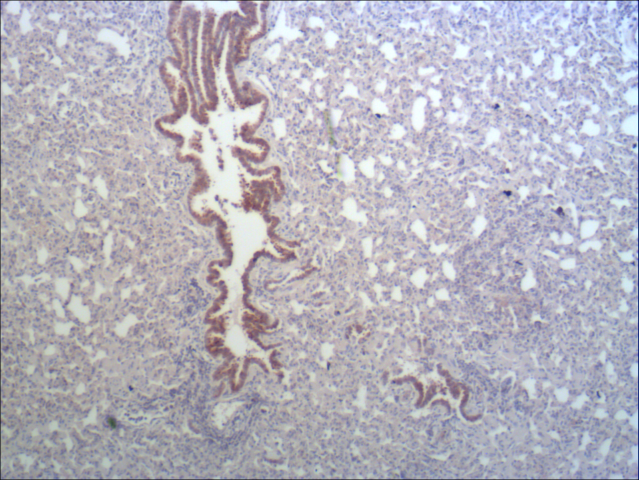* | **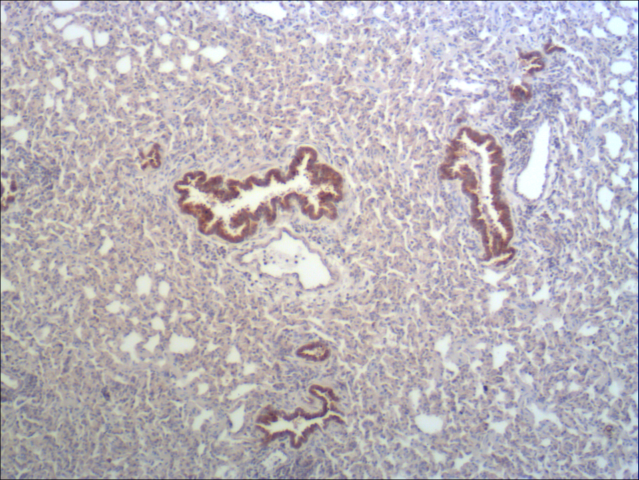** |

| ***D)***  ***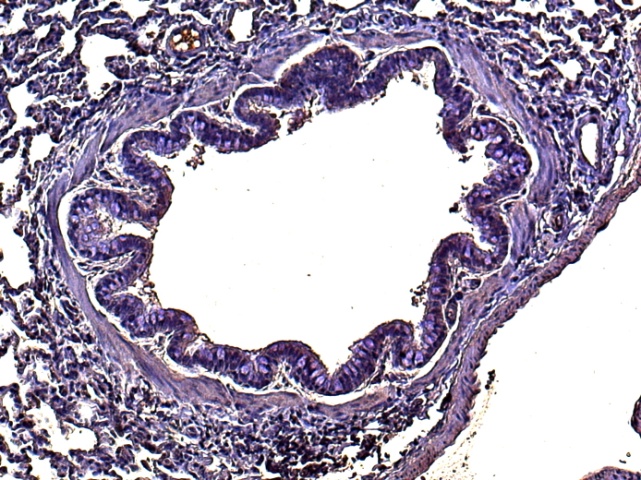*** | **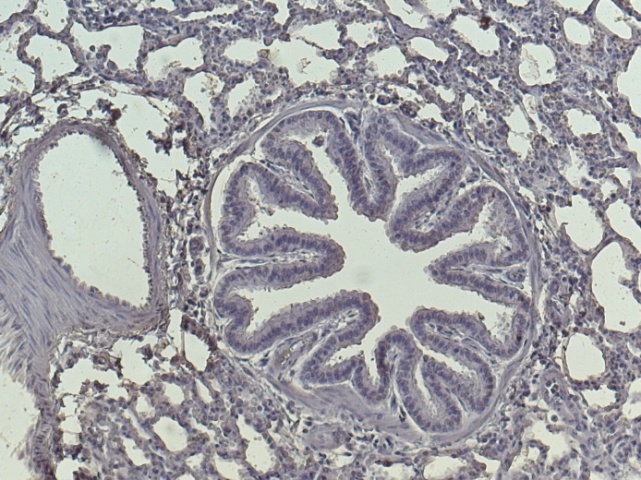** |
| --- | --- |
| ***E)***  ***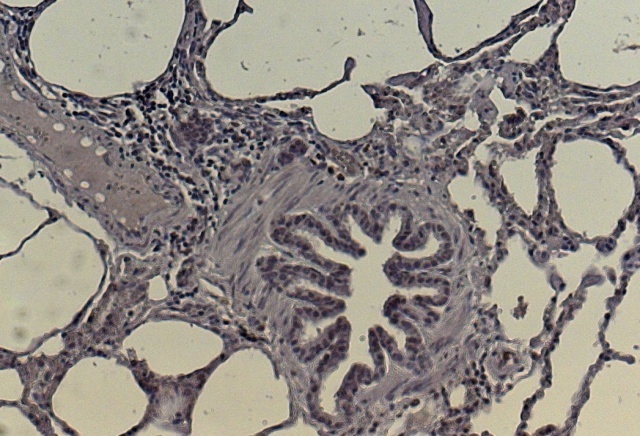*** | **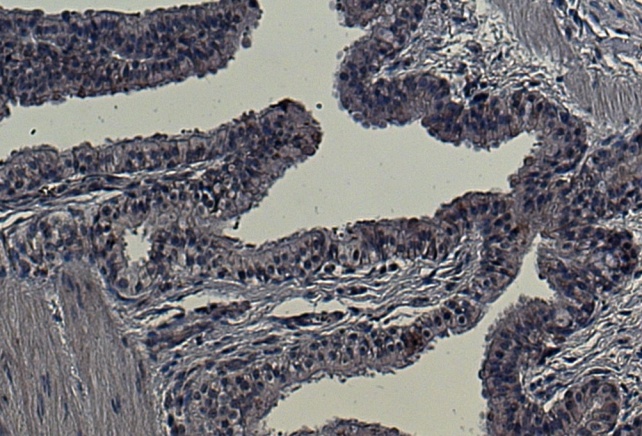** |
| ***F)***  ***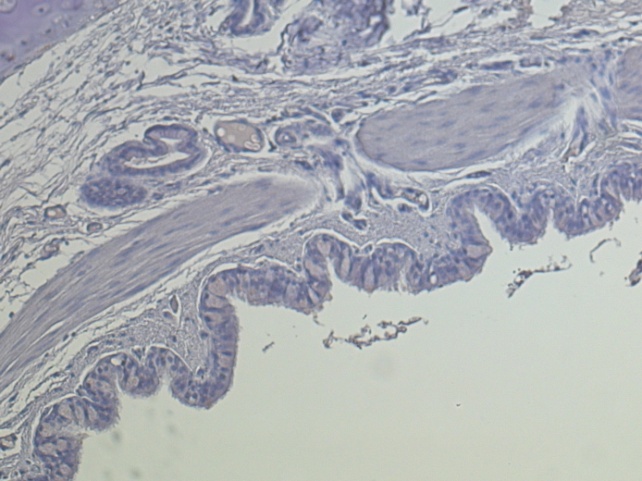*** | **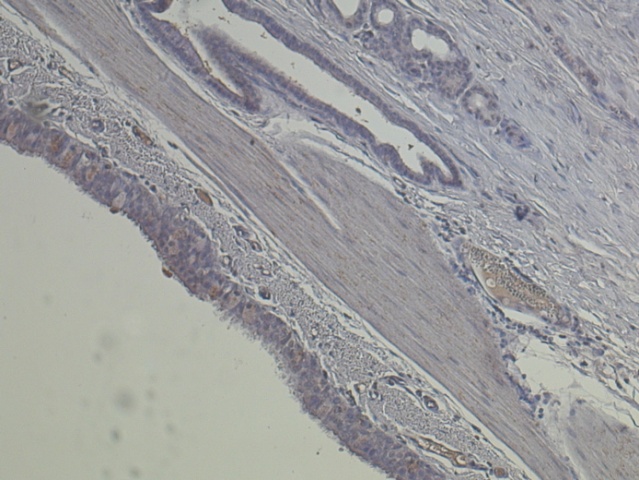** |
| ***G)***  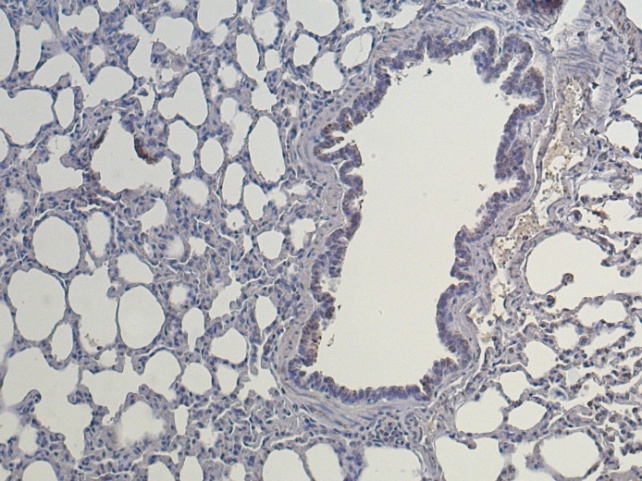 | **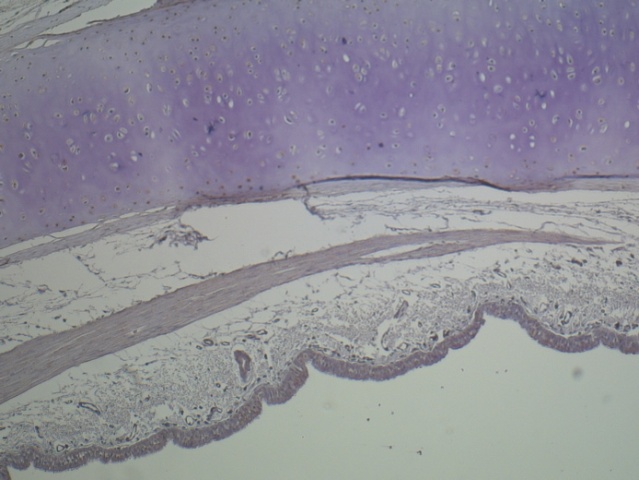** |

*The left column represents Control animals. The right column represents Brain Dead animals. A) ET-1. B) ET_R_A. C) ET_R_B. D) MMP-2. E) MMP-9. F) TIMP-1. G) TIMP-2*
